# Variable intraspecific response to climate change in a medicinally important African tree species, *Vachellia sieberiana* (DC.) (paperbark thorn)

**DOI:** 10.1002/ece3.11314

**Published:** 2024-04-29

**Authors:** Percy Jinga, Tawanda Manyangadze

**Affiliations:** ^1^ Biological Sciences Department Bindura University of Science Education Bindura Zimbabwe; ^2^ Geosciences Department Bindura University of Science Education Bindura Zimbabwe

**Keywords:** climate change, conservation, ensemble species distribution model, shared socioeconomic pathway, species variety, *Vachellia sieberiana*

## Abstract

Climate change is predicted to disproportionately impact sub‐Saharan Africa, with potential devastating consequences on plant populations. Climate change may, however, impact intraspecific taxa differently. The aim of the study was to determine the current distribution and impact of climate change on three varieties of *Vachellia sieberiana*, that is, var. *sieberiana*, var. *villosa* and var. *woodii*. Ensemble species distribution models (SDMs) were built in “biomod2” using 66, 45, and 137 occurrence records for var. *sieberiana*, var. *villosa*, and var. *woodii*, respectively. The ensemble SDMs were projected to 2041–2060 and 2081–2100 under three general circulation models (GCMs) and two shared socioeconomic pathways (SSPs). The three GCMs were the Canadian Earth System Model version 5, the Institut Pierre‐Simon Laplace Climate Model version 6A Low Resolution, and the Model for Interdisciplinary Research on Climate version 6. The suitable habitat of var. *sieberiana* predominantly occurs in the Sudanian and Zambezian phytochoria while that of var. *villosa* largely occurs in the Sudanian phytochorion. The suitable habitat of var. *woodii* mainly occurs in the Zambezian phyotochorion. There is coexistence of var. *villosa* and var. *sieberiana* in the Sudanian phytochorion while var. *sieberiana* and var. *woodii* coexist in the Zambezian phytochorion. Under SSP2‐4.5 in 2041–2060 and averaged across the three GCMs, the suitable habitat expanded by 33.8% and 119.7% for var. *sieberiana* and var. *villosa*, respectively. In contrast, the suitable habitat of var. *woodii* contracted by −8.4%. Similar trends were observed in 2041–2060 under SSP5‐8.5 [var. *sieberiana* (38.6%), var. *villosa* (139.0%), and var. *woodii* (−10.4%)], in 2081–2100 under SSP2‐4.5 [var. *sieberiana* (4.6%), var. *villosa* (153.4%), and var. *woodii* (−14.4%)], and in 2081–2100 under SSP5‐8.5 [var. *sieberiana* (49.3%), var. *villosa* (233.4%), and var. *woodii* (−30.7%)]. Different responses to climate change call for unique management and conservation decisions for the varieties.

## Graphical abstract

Three varieties of *Vachellia sieberiana* respond differently to climate change. The suitable habitat of two varieties, that is, var. *villosa* and var. *sieberiana*, expands when forecasted under three general circulation models and two shared socioeconomic pathways. In contrast, the suitable habitat of var. *woodii* contracts.

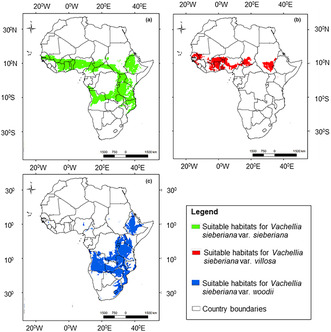

## 1 INTRODUCTION

Climate change, primarily caused by anthropogenic emission of greenhouse gases, has resulted in mean global surface temperature reaching 1.1°C above 1850–1900 level in 2011–2020 (IPCC, 2023). Mean global surface temperature increase is altering precipitation patterns and causing substantial changes in ecosystem structure, species ranges, and seasonal timing (Büntgen et al., 2022; Esperon‐Rodriguez et al., 2022; Stemkovski et al., 2023). Vulnerability to climate change varies globally, with communities that have historically contributed the least greenhouse gases, such as sub‐Saharan Africa, being disproportionately affected (Affoh et al., 2022; IPCC, 2023; Kompas et al., 2018; Sintayehu, 2018). Mean surface temperatures have increased by 0.7°C in sub‐Saharan Africa in the 20th century, and warming is expected to increase by 0.2–0.5°C per decade in the 21st century (Sintayehu, 2018). As the impacts of climate change are expected to be more consequential before mitigation results are realized, biodiversity loss, species range changes, and loss of ecosystem services may likely become more apparent. The potential future disappearance of especially some sub‐Saharan African plant species is catastrophic to 60%–70% of economically vulnerable rural populations that directly depend on indigenous plant resources for their livelihood (Güneralp et al., 2017). It is, thus, crucial to understand the impact of climate change on the distribution and abundance of sub‐Saharan African plant species that sustain so many vulnerable livelihoods.

The impact of climate change on the distribution and abundance of species is now commonly investigated by species distribution models (SDMs). SDMs determine a statistical relationship between a set of environmental variables and a species' geographic distribution represented by occurrence records (Elith & Leathwick, 2009; Guisan & Zimmermann, 2000). The statistical relationship is then applied to different geographic areas to determine probability of occurrence (Guisan & Thuiller, 2005), and to different greenhouse gas emission scenarios to determine future changes of climatically suitable habitats (Pearson & Dawson, 2003). Although SDMs have some shortcomings, such as the failure to incorporate dispersion, competition, and other biotic interactions in predicting the distribution of species (Grimmett et al., 2021; Moens et al., 2022; Wisz et al., 2013), they have been effectively and widely used in conservation planning (Rathore & Sharma, 2023), determining the present and future distributions of species under climate change (Franklin, 2023), and in investigating evolutionary processes (Kozak et al., 2008; Razgour et al., 2019).

Species distribution models have commonly been used to predict the distribution and response to climate change at the species level with a generalization that intraspecific taxa respond in a similar trend. The assumption that intraspecific taxa respond similarly to climate change is made despite strong evidence of local adaptation in plants (Baughman et al., 2019; van Boheemen et al., 2019). Plants experience local biotic interactions and microenvironments that shape their evolution. Intraspecific taxa occurring over wide geographic areas and experiencing different biotic and abiotic conditions are likely to develop variable life‐history traits over time. Species‐level SDMs used to predict the potential distribution and response to climate change for widespread species can result in some intraspecific taxa having little representation in the resultant models (D'Amen et al., 2013; DeMarche et al., 2019; Jinga et al., 2021). The absence of intraspecific information in SDMs may result in failure to detect threats, and thus, inappropriate conservation recommendations. Incorporation of intraspecific information in SDMs has recently been attempted to improve niche characterization and to identify responses to climate change that would otherwise not be detected by species‐level SDMs (Chardon et al., 2020; D'Amen et al., 2013; Pearman et al., 2010; Serra‐Varela et al., 2015). Most of the studies, however, that incorporated intraspecific information into SDMs have been done for Australian (Thompson et al., 2011; Tingley et al., 2016), European (Chardon et al., 2020; Pearman et al., 2010; Serra‐Varela et al., 2015), and North American (Bemmels et al., 2016; Chardon et al., 2020; Schwalm et al., 2016; Shinneman et al., 2016) species. In this study, we aimed to estimate the current distribution of three varieties of *V. sieberiana* and determine the impact of climate change after incorporation of intraspecific information in ensemble SDMs. In addition to investigating the impact of climate change at the sub‐specific level, SDMs were also used to identify areas of niche overlap among varieties of *V. sieberiana*.

*Vachellia sieberiana* (DC.) (paperbark thorn), formerly *Acacia sieberiana* (DC.), a sub‐Saharan Africa tree species, encompasses three varieties, which are var. *sieberiana*, var. *villosa*, and var. *woodii* (Kyalangalilwa et al., 2013). Two of the varieties are distinguished by the median leaflet size, with var. *villosa* having 2.0–2.5‐mm‐long leaflets and var. *sieberiana* with 2.5–4.5 mm (Keay & Brenan, 1950). Variety *woodii* has variable leaflet size but can be distinguished from the other two varieties by a much coarser shoot indumentum that is villous and golden, and a fruit that is densely pubescent when young (Keay & Brenan, 1950). Extracts from *V. sieberiana* bark, leaf, and root have been traditionally used to treat bilharzia, gonorrhea, stomach aches, urethral infections, and diarrhea, among several illnesses (Orwa et al., 2003). Antimicrobial effects of some *A. sieberiana* extracts have been reported on *Mycobacterium aurum*, *Bacillus subtilis*, *Staphylococcus aureus*, *Escherichia coli*, *Klebsiella pneumoniae*, *Salmonella typhi*, *Helicobacter pylori*, *Shigella dysenteriae*, and *Staphylococcus epidermis* (Eldeen et al., 2005; Eldeen & Van Staden, 2007; Kirabo et al., 2018; Rabe & Van‐Staden, 1997). The presence of *V. sieberiana* leaf extracts with cytotoxic effect against multidrug‐resistant cancer cells has been an important discovery (Ngaffo et al., 2020, 2022) that has generated interest in studying other likely medicinal benefits, the ecology and natural distribution of the species. The species is categorized under “Least Concern” by the IUCN because of its wide geographic distribution and a comparatively large population size in sub‐Saharan Africa.

The comprehensive natural distributions of the three varieties are not well defined despite the medicinal importance of the species. The impact of climate change on the natural distributions is also important to study since sub‐Saharan Africa will potentially be disproportionately affected. The objectives of the study were to (i) establish the current natural distribution of var. *sieberiana*, var. *villosa*, and var. *woodii*, (ii) determine areas of coexistence of the varieties, and (iii) forecast changes in size of the suitable habitat of the varieties under different climate change scenarios. Current distribution maps are important in guiding botanical explorations, identifying sampling areas, and providing spatial information for conservation. Determining the impact of climate change at intraspecific level may reveal variable responses, resulting in different management and conservation recommendations that are specific and more effective for each intraspecific taxon.

## 2 MATERIALS AND METHODS

### 2.1 Species occurrence records

Occurrence records of the three varieties of *V. sieberiana* were obtained from the Global Biodiversity Information Facility (GBIF; https://www.gbif.org; GBIF, 2023a, 2023b, 2023c) and the RAINBIO (Dauby et al., 2016) online data portals. The GBIF data are especially comprehensive because, apart from having occurrence records drawn from professional databases, they also include those collected by citizen science tools and not readily available in other forest inventories (Dyderski et al., 2018). Automatic filters on the GBIF portal were used to screen potentially erroneous occurrence records, such as those with invalid geodetic data, coordinates out of range, and presumed swapped coordinates. RAINBIO is a compilation of 13 datasets focusing on vascular plants in continental tropical Africa, particularly south of the Sahel and north of southern Africa.

**FIGURE 1:**
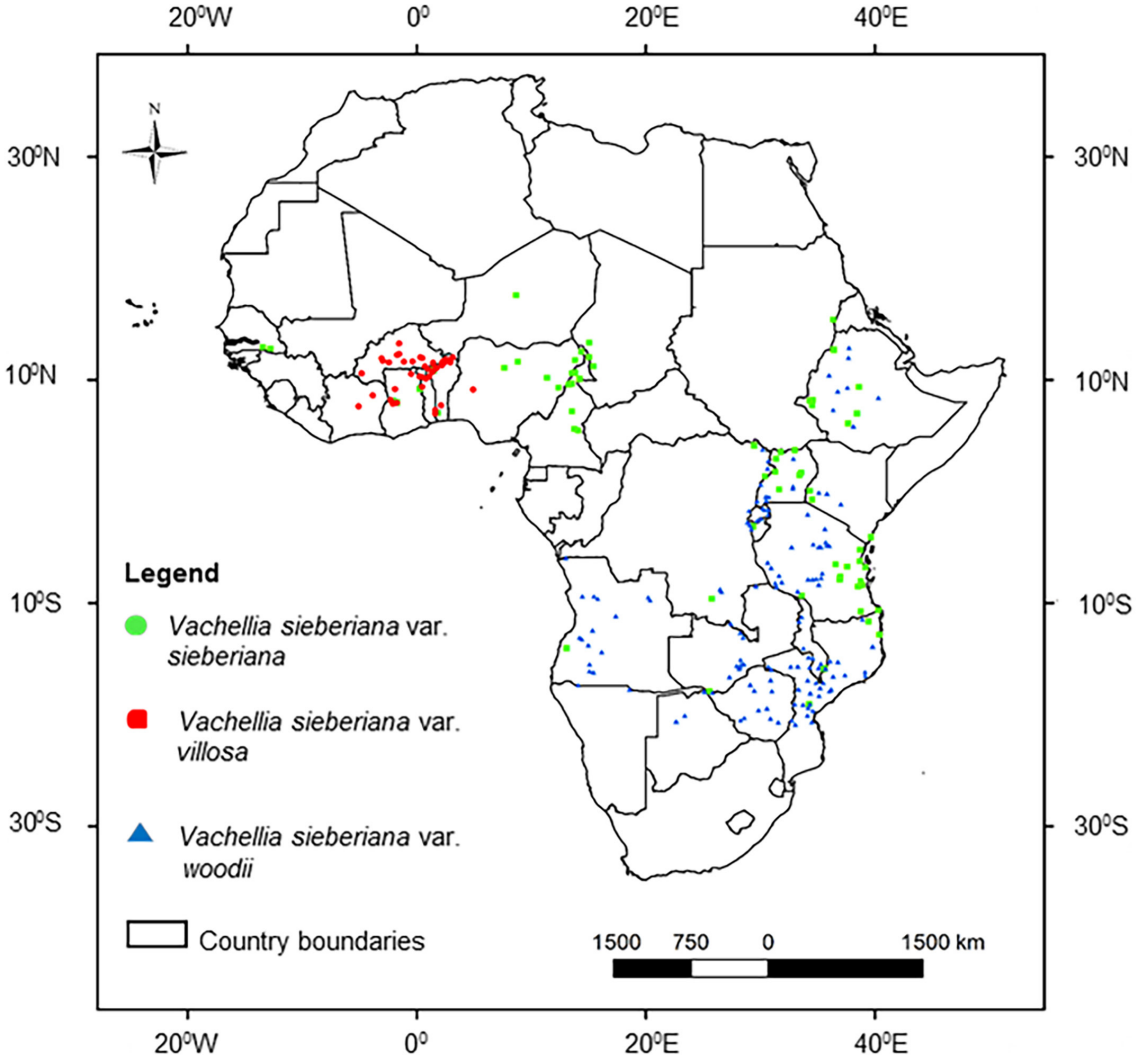
Location of occurrence records of three varieties of *Vachellia sieberiana*, namely var. *sieberiana*, var. *villosa*, and var. *woodii*, which were used in ensemble species distribution models to estimate current distributions and the impact of climate change.

The occurrence records span almost the entire sub‐Saharan Africa, from the Sahel in the north to the savanna of southern Africa, showing that *V. sieberiana* is a widespread tree species (Figure 1). The range of the species occurs in some major tropical climate regions, including the semi‐arid in the Sahel and the grasslands of southern Africa. Major vegetation types found in the natural range of *V. sieberiana* include the eastern and southern African lowland evergreen and semi‐evergreen forest, the Sudano‐Sahelian dry savanna, and the Guineo‐Congolian semi‐deciduous rainforest (Sayre et al., 2013). Various soil types are found in the range of *V. sieberiana*, including plinthosols and lixisols north of the equator, as well as cambisols and luvisols in eastern and southern Africa (Dewitte et al., 2013). The widespread occurrence of *V. sieberiana* in different soil and climate types suggests that the species is a generalist.

After removing outliers and duplicates, totals of 164, 128, and 361 occurrence records were obtained for var. *sieberiana*, var. *villosa*, and var. *woodii*, respectively. The occurrence records showed biased sampling, with high‐intensity sampling in some areas (Appendix S1). Uneven sampling causes overrepresentation of environmental features of the extensively sampled areas, leading to inaccurate models (Kramer‐Schadt et al., 2013). Significance may be attributed to environmental variables that are typical of the areas with clumped occurrence records, resulting in spatial extrapolation errors. These errors are omission errors or false negatives, where a species is mistakenly concluded to be absent, and commission errors or false positives, where a species is mistakenly concluded to be present (Rondinini et al., 2006). Models generated with spatially biased occurrence records may lead to inappropriate management decisions.

To correct spatial bias, occurrence records of the varieties were resampled in R (www.r‐project.org/) (R Core Team, 2023) using the package “spThin” (Aiello‐Lammens et al., 2015). The resampling process in “spThin” takes a set of occurrence records and generates several new datasets that meet a user‐specified minimum nearest‐neighbor distance (NND) constraint. The NND constraint is the minimum distance between occurrence records that will be maintained in the output datasets (Aiello‐Lammens et al., 2015). After testing several NND constraints, a minimum distance of 20 km between occurrence records showed relatively uniform distributions across all three study landscapes. After the resampling process, 66, 45, and 137 occurrence records were used for model calibration and projections for var. *sieberiana*, var. *villosa*, and var. *woodii*, respectively (Figure 1).

### 2.2 Environmental variables

Bioclimatic variables (Booth et al., 2014) and elevation obtained from the WorldClim version 2.1 online data portal (https://worldclim.org) (Fick & Hijmans, 2017) were used as environmental variables in model calibration. Bioclimatic variables are derived from precipitation and temperature. They describe seasonal or annual variations in temperature and precipitation, and represent climate extremes that are more limiting to plant distribution than monthly climate variables (Bede‐Fazekas & Somodi, 2020; Watling et al., 2012). Bioclimatic variables are more ecologically and physiologically relevant to plant growth and establishment (Title & Bemmels, 2018), and SDMs generated from them should be more robust (Araújo & Guisan, 2006; Austin, 2002). As a result, bioclimatic variables have been widely used in SDMs to determine conservation areas for imperiled species (Malakoutikhah et al., 2020; Varela et al., 2022), predict the impact of climate change on the distribution and abundance of species (Balima et al., 2022; Esser & Weldemariam, 2023), and to establish current geographic distributions (Hama & Khwarahm, 2023).

Several methods are available to determine environmental variables to use in SDMs, each with advantages and disadvantages. Variables can be selected according to expert ecological knowledge on how relevant they are to the focal species (Amoussou et al., 2022; Blach‐Overgaard et al., 2015). Variables can be selected by permutation tests implemented in SDM algorithms, where those with high variable importance scores are selected (Braunisch et al., 2013). Variables can also be selected after correlation analysis (Elith et al., 2006). Variables that are correlated decrease model transferability, increase overfitting, decrease signal‐to‐noise ratio, increase computation time, and result in inaccurate interpretation of causal relationships (De Cauwer et al., 2014; Jarnevich et al., 2015). In this study, variables with a Pearson's correlation coefficient (*r*) of less than 0.70 (Dormann et al., 2013; Jarnevich et al., 2015) and a variance inflation factor (VIF) of less than 10 (O'Brien, 2007; Steen et al., 2017) were selected in R using the “usdm” package (Naimi et al., 2014) for the three varieties. Six variables were selected for var. *sieberiana* and var. *villosa* while seven were selected for var. *woodii* (Table 1). All variables were downloaded at a spatial resolution of 2.5 min.

**TABLE 1:** Importance scores of environmental variables used in ensemble species distribution models of three varieties of *Vachellia sieberiana*.

Variety	Variable	Importance score
Var. *sieberiana*	BIO2	0.025
	BIO4	0.021
	BIO9	0.261
	BIO12	0.183
	BIO13	0.251
	Elevation	0.215
Var. *villosa*	BIO4	0.035
	BIO8	0.002
	BIO11	0.314
	BIO16	0.014
	BIO19	0.011
	Elevation	0.128
Var. *woodii*	BIO2	0.045
	BIO4	0.022
	BIO9	0.041
	BIO12	0.194
	BIO13	0.027
	BIO18	0.070
	Elevation	0.303

*Note*: BIO2 = annual mean temperature, BIO4 = temperature seasonality, BIO8 = mean temperature of wettest quarter, BIO9 = mean temperature of driest quarter, BIO11 = mean temperature of coldest quarter, BIO12 = annual precipitation, BIO13 = precipitation of wettest quarter, BIO16 = precipitation of wettest quarter, BIO18 = precipitation of warmest quarter, BIO19 = precipitation of coldest quarter.

### 2.3 Calibration and ensembling of models

Individual models were calibrated and ensembled in “biomod2” version 4.2‐4 (Thuiller, 2003; Thuiller et al., 2009, 2023). “Biomod2” is an R‐based software that can combine up to 12 different modeling algorithms into an ensemble model for prediction of species distributions. In this study, seven algorithms were used, and these are artificial neural network (Ripley, 2007), classification tree analysis (Breiman et al., 1984), flexible discriminant analysis (Hastie et al., 1994), generalized boosted model (Friedman, 2001), generalized linear model (McCullagh & Nelder, 1989), multivariate adaptive regression splines (Friedman, 1991), and random forest (Breiman, 2001).

To calibrate the seven individual models, 66, 45, and 137 pseudoabsences were selected for var. *sieberiana*, var. *villosa*, and var. *woodii*, respectively. The number of pseudoabsences selected was equal to the number of true presences for each of the varieties. Equal numbers of true presences and pseudoabsences were used to avoid imbalances in evaluating model performance during calibration (Japkowicz & Stephen, 2002). The “disk” function in “biomod2” was used to specify a minimum distance of 20 km from a true presence where a pseudoabsence can be located. The “disk” method was preferred to the complete random method because it eliminates the chance of placing pseudoabsences where true presences might have been located but removed during resampling. Occurrence records were split into 30% for testing and 70% for training, with a 10‐fold cross‐validation to evaluate the individual models for the three varieties. Four metrics were used to evaluate the individual models, namely area under the receiver operating characteristic curve (ROC) (Hanley & McNeil, 1982), Cohen's kappa (KAPPA) (Cohen, 1960), true skill statistic (Allouche et al., 2006), and overall accuracy (Accuracy) (Finley, 1884).

An important feature of “biomod2” is the ability to combine individual SDMs into an ensemble model using a threshold evaluation score. In this study, individual SDMs with an ROC score above 0.90 were combined into an ensemble model. The SDMs were weighted according to their evaluation score. Weighting the SDMs has been shown to improve overall ensemble model accuracy compared to other methods of combining them (Hao et al., 2019; Marmion et al., 2009). The ensemble model for each variety was used to estimate the current distribution as well as projections under different climate change scenarios. An ROC threshold implemented in “biomod2” was used to convert continuous distribution maps into binary presence/absence maps.

### 2.4 Forecasting

The ensemble model for each variety was projected for years 2041–2060 and 2081–2100 under two shared socioeconomic pathways (SSPs) and three general circulation models (GCMs). SSPs are a new generation of future pathways that determine the level of greenhouse gas emissions by taking into consideration population and economic growth, education, urbanization, and the rate of technological development. The two SSP scenarios used in this study are SSP2‐4.5 and SSP5‐8.5. Under SSP2‐4.5, the fossil fuel energy sector continues to play a major role until 2080, while under SSP5‐8.5, greenhouse gases continue to be produced throughout the 21st century caused by resource‐intensive lifestyles sustained by fossil fuel development (Gidden et al., 2019; Riahi et al., 2017). The two SSPs were selected in order to capture a wide range of possibilities on the effects of climate change, from the optimistic to the pessimistic.

General circulation models evaluate the response of climate to greenhouse gas concentration due to long‐term trends in energy and land use (Rogelj et al., 2012). The performance of SDMs is substantially affected by the choice of GCMs (Goberville et al., 2015). GCMs vary due to a host of factors, such as different parameterizations of natural processes and use of different spatial resolutions (Timbal, 2004). It is, thus, advisable to use several GCMs to cater to inter‐GCM variability. The GCMs used in this study were the Canadian Earth System Model version 5 (CanESM5) (Swart et al., 2019), the Institut Pierre‐Simon Laplace Climate Model version 6A Low Resolution (IPSL‐CM6A‐LR) (Boucher et al., 2020), and the Model for Interdisciplinary Research on Climate version 6 (MIROC6) (Tatebe et al., 2019). The selected GCMs have been widely used in SDMs to make predictions of species' occurrence and impact of climate change (e.g., Adenkanmbi et al., 2023; Chen et al., 2023; Mallen‐Cooper et al., 2023). The GCMs were selected by maximizing the intermodel distance among them according to Sanderson et al. (2015).

## 3 RESULTS

### 3.1 Current distributions

The ensemble models showed robust performance, with most ROC and accuracy scores above 0.90 and 0.80, respectively (Table 2). Elevation contributed significantly to all three ensemble models (Table 1). Response curves show that the optimum elevation ranges are 1000–2000 m for var. *sieberiana* and var. *woodii*, and 100–300 m for var. *villosa* (Appendix S2). Thus, var. *sieberiana* and var. *woodii* are comparatively high‐altitude varieties while var. *villosa* is a low‐altitude variety. Apart from elevation, annual mean precipitation also contributed significantly to the ensemble model of var. *sieberiana* and var. *woodii*. The optimum annual mean precipitation range for var. *sieberiana* and var. *woodii* is 750–1250 mm while the optimum precipitation of the wettest quarter for var. *villosa* is about 500 mm (Appendix S2). Mean temperature of the coldest quarter is also important for the distribution of var. *villosa*, with temperatures above 25°C being optimum (Appendix S2). The results indicate that var. *villosa* may be the most resistant to dry conditions among the three varieties.

**TABLE 2:** Scores of four metrics used to evaluate ensemble species distribution models developed for three varieties of *Vachellia sieberiana*.

	Evaluation metric			
	Accuracy	KAPPA	ROC	TSS
Var. *sieberiana*	0.813	0.596	0.869	0.621
Var. *villosa*	0.941	0.870	0.987	0.889
Var. *woodii*	0.844	0.650	0.914	0.690

**FIGURE 2:**
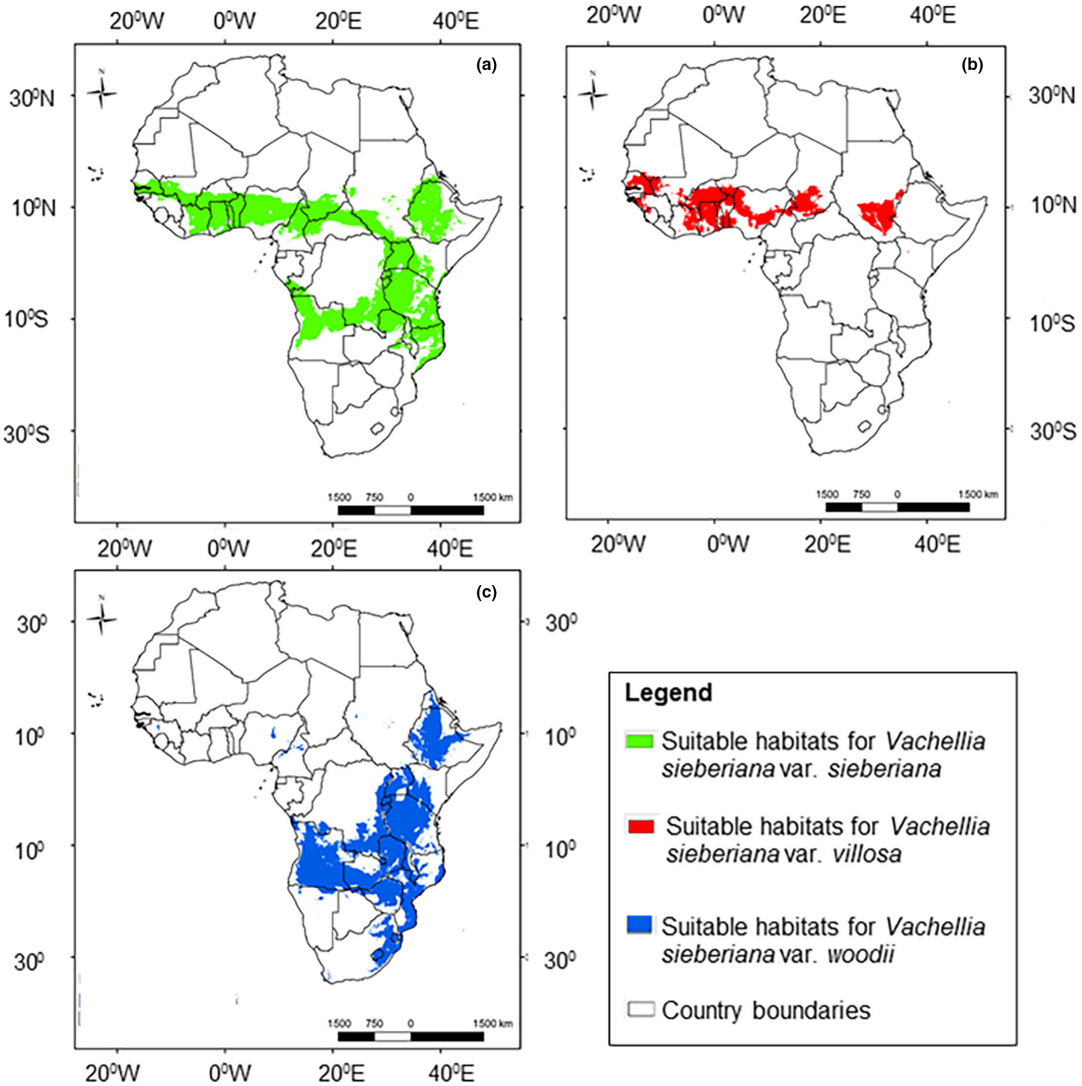
Current natural distribution in sub‐Saharan Africa of three varieties of *Vachellia sieberiana*, which are var. *sieberiana* (a), var. *villosa* (b), and var. *woodii* (c), estimated by ensemble species distribution models.

**FIGURE 3:**
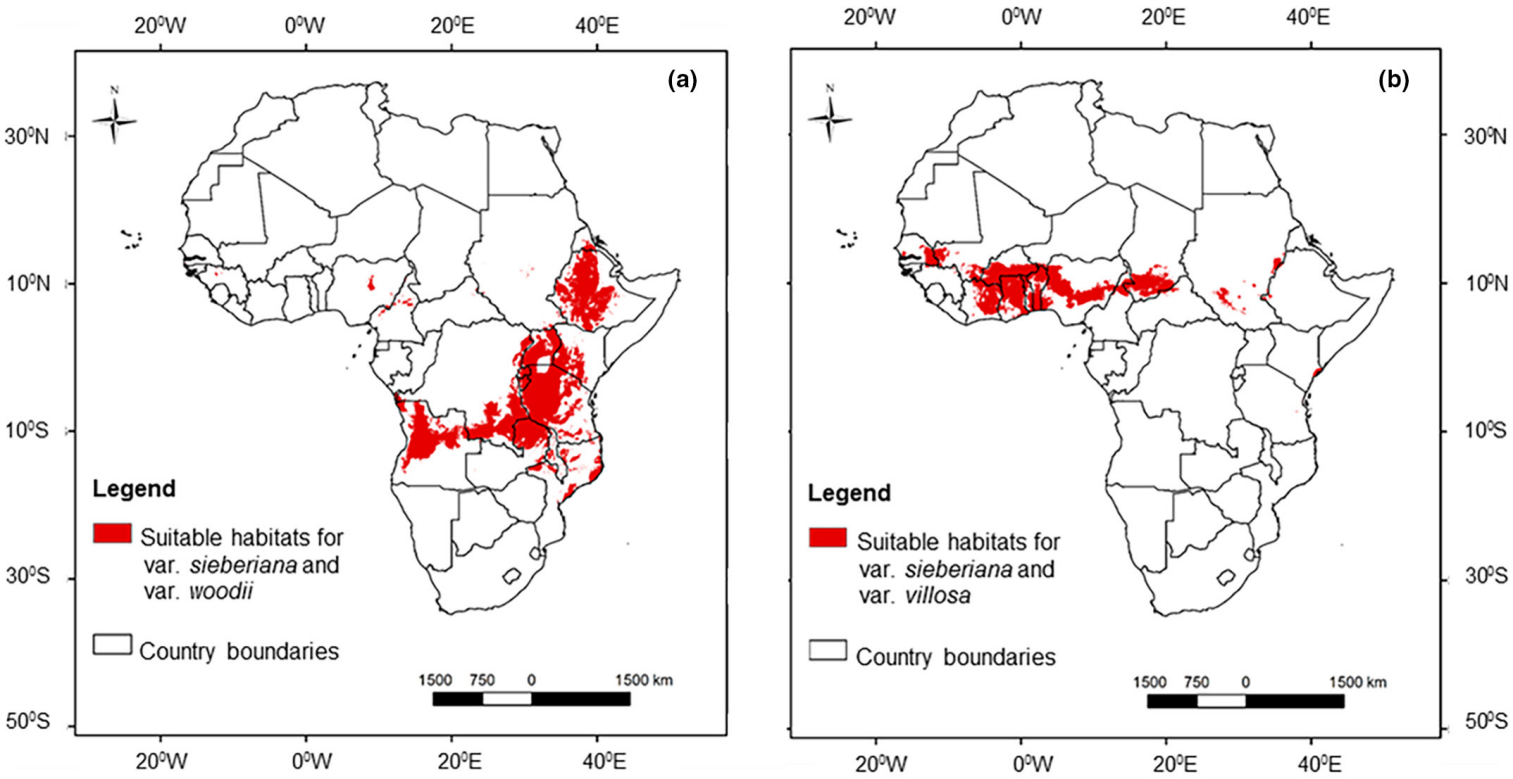
Coexistence ranges of varieties of *Vachellia sieberiana*, that is, var. *sieberiana* and var. *woodii* (a), and var. *sieberiana* and var. *villosa* (b), which were estimated by ensemble species distribution models.

Using White (1983)'s classification of African vegetation, the suitable habitat of var. *sieberiana* currently occurs in the Sudanian, Zambezian, and Afromontane/Alpine phytochoria (Figure 2a). The habitat stretches from Senegal in West Africa to Ethiopia, Uganda, and Kenya in East Africa. In southern Africa, the suitable habitat of var. *sieberiana* is located in Angola, northern Mozambique, Malawi, and Zambia. The suitable habitat of var. *villosa* is the smallest among the three varieties and is restricted north of the equator, predominantly in the Sudanian phytochorion (Figure 2b). The suitable habitat of var. *villosa* is found in Senegal, southern Mali, Burkina Faso, Benin, Togo, Ghana, Nigeria, South Sudan, and southern Chad. Variety *woodii* is naturally distributed in east and southern Africa, in the Afromontane/Alpine and Zambezian phytochoria (Figure 2c). In southern Africa, var. *woodii* is distributed in South Africa, Zimbabwe, Angola, Zambia, Mozambique, and Malawi. In East Africa, the suitable habitat of var. *woodii* occurs in Ethiopia, Kenya, Tanzania, Uganda, Rwanda, and Burundi. Results show that var. *sieberiana* and var. *woodii* coexist in the Zambezian and Afromontane/Alpine phytochoria (Figure 3a). Coexistence was also observed of var. *sieberiana* and var. *villosa* north of the equator in the Sudanian phytochorion (Figure 3b). There was, however, no habitat found to be suitable for coexistence of all three varieties.

### 3.2 Future distributions

**FIGURE 4:**
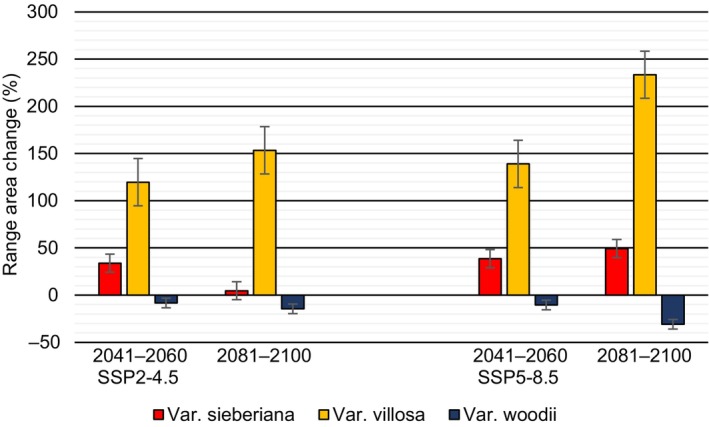
Predicted changes in size of suitable habitat of three varieties of *Vachellia sieberiana* under climate change averaged across three general circulation models for two shared socioeconomic pathways and two time periods, 2041–2060 and 2081–2100.

Averaged across three GCMs, projections showed the suitable habitat of var. *sieberiana* and var. *villosa* expanding while that of var. *woodii* contracting under all SSP scenarios and time periods (Figure 4). In 2041–2060 under SSP2‐4.5, the suitable habitat expanded by 33.8% and 119.7% for var. *sieberiana* and var. *villosa*, respectively, while that for var. *woodii* contracted by −8.4%. In 2041–2060 under SSP5‐8.5, the expansions were 38.6% and 139.0% for var. *sieberiana* and var. *villosa*, respectively, and a contraction of −10.4% for var. *woodii* (Figure 4). A similar trend was observed in 2081–2100 under SSP2‐4.5, showing the expansion of the natural range of var. *sieberiana* (4.6%) and var. *villosa* (153.4%), and a contraction for var. *woodii* (−14.4%). The highest expansions were observed in 2081–2100 under SSP5‐8.5 of 49.3% and 233.4% for var. *sieberiana* and var. *villosa*, respectively. The highest contraction for var. *woodii* (−30.7%) was also observed in 2081–2100 under SSP5‐8.5. The three GCMs showed similar trends, either contraction or expansion, for all varieties, time periods, and SSP scenarios (Appendix S3).

The suitable habitat of var. *sieberiana* was predicted to expand under climate change into the Sahel and Somali‐Masai phytochoria (Appendix S4). The suitable habitat of var. *sieberiana* was also shown to expand further south in the Zambezian phytochorion. For var. *villosa*, the suitable habitat expands from the Sudanian phytochorion into the Sahel and the Guinea‐Congolia/Sudania transition. Contraction from the southern edges of the Zambezian phytochorion and in the Afromontane/Afroalpine phytochorion was observed for var. *woodii* (Appendix S4). There was little variability in the areas of expansion and contraction across the three GCMs for each variety.

## 4 DISCUSSION

Current distribution maps of var. *woodii* in southern Africa have been produced (e.g., Palgrave, 2002; Van Wyk et al., 2011; Van Wyk & Van Wyk, 1997). However, the current distribution maps do not cover the entire natural range where var. *woodii* has been sampled and known to occur. The current distribution maps of var. *sieberiana* and var. *villosa* have been shown as entire countries in which the varieties have been observed and or sampled (POWO, 2023). General country maps, however, have the drawback of showing presence across the entire country although a species may be absent in some parts of the country. Our study represents the first attempt to provide maps showing the entire natural ranges of all three varieties. The baseline current distribution maps in this study are especially important in the current period of rapid and largely anthropogenic climate change as they provide reference points against which future changes can be measured when calculating extinction risk (Gaston & Fuller, 2009; Jelaska, 2009). The current distribution maps are also vital for selecting areas for conservation and or preservation, determining forest management practices, informing researchers on potential sampling areas, and providing baseline information to botanical explorers.

Several theories have been proposed to explain coexistence in plants, each with critics and supporters alike. These theories of plant coexistence include, among several, coexistence maintained by niche differentiation (Gause, 1934), and coexistence without niche differentiation (Aarssen, 1983, 1984; Zobel, 1992). The niche differentiation theory has been deemed inadequate to explain the high plant species richness, especially in the tropics (Cole, 1960; Hulme, 1996; Walter, 1988). Plants are largely immobile and lack the ability to move in search of resources or to escape from predators. Thus, if they coexist, they are likely to be ecologically and functionally similar, that is, their niches overlap (Kim & Ohr, 2020). The ecological and functional similarities imply comparable morphology, dispersion, growth rate, height, and seed production rate, hence similar competition for resources of the coexisting plants (Kim & Ohr, 2020). The coexistence of var. *villosa* and var. *sieberiana* in the Sudanian and Sahel phytochoria suggests ecological and functional similarity. Unsurprisingly, it has been suggested that var. *villosa* was derived from a small‐leafleted form of var. *sieberiana* (Keay & Brenan, 1950). Similarly, the coexistence of var. *woodii* and var. *sieberiana* in the Zambezian phytochorion has led to the suggestion that var. *woodii* is a variant of var. *sieberiana* with a more extreme development of the indumentum (Keay & Brenan, 1950). Thus, var. *sieberiana* may be considered to be functionally and ecologically intermediate between var. *villosa* and var. *woodii*. Coexistence makes it important and necessary in studies of *V. sieberiana* to identify the species to variety level, such as when investigating presence of phytochemicals with medicinal properties.

Different responses to climate change of intraspecific taxa have been reported in a broad range of organisms, including algae (Assis et al., 2016), insects (Homburg et al., 2014; Lecocq et al., 2016; Meynard et al., 2017), birds (Liu et al., 2012; Strubbe et al., 2015), reptiles (Tingley et al., 2016), and tree species (Hu et al., 2017; Ikeda et al., 2017; Jinga et al., 2021). SDMs that overlook intraspecific information obscure the often different niches occupied by intraspecific taxa and assume similar responses to climate change (Pearman et al., 2010). Species‐level SDMs may smooth environmental response curves of especially intraspecific taxa restricted in distribution and highly adapted to a specific set of climatic conditions (Chardon et al., 2020). Restricted intraspecific taxa may contribute few occurrence records and have little influence on a species‐level SDM (Elith & Leathwick, 2009; Osborne et al., 2007). However, the restricted intraspecific taxa may have unique responses to climate change that are critical for management decisions and may be missed by species‐level SDMs (Jinga et al., 2021). Fine taxonomic resolution in SDMs is vital to reveal unique responses to climate change.

In this study, it was shown that the three *V. sieberiana* varieties respond differently to climate change, with the suitable habitat of var. *woodii* contracting while that of var. *sieberiana* and var. *villosa* expanding. Our results are consistent with other studies showing different responses to climate change of intraspecific taxa that are geographically disparate (e.g., Chardon et al., 2020; Hu et al., 2017; Ikeda et al., 2017; Serra‐Varela et al., 2017). By 2030–2059, the mean annual temperature under several GCMs across the African continent is projected to increase by 1.2, 1.5, and 1.8°C under SSP1‐2.6, SSP2‐4.5, and SSP5‐8.5, respectively (Almazroui et al., 2020). In the long‐term, that is, 2070–2099, the mean annual temperature is projected to increase by 1.4, 2.3, and 4.4°C under SSP1‐2.6, SSP2‐4.5, and SSP5‐8.5, respectively (Almazroui et al., 2020). While temperatures will generally increase across the entire African continent, annual precipitation trends show regional variability. In southern Africa, where var. *woodii* predominates, annual precipitation is projected to decline (Almazroui et al., 2020; Dosio et al., 2021). In contrast, however, annual precipitation is projected to increase in West Africa where var. *villosa* predominates (Almazroui et al., 2020; Dosio et al., 2021). Amount of water available for uptake has a profound effect on growth and development, especially for those plants that lack xeromorphic features.

The contraction of the suitable habitat of var. *woodii* in future is likely in response to declining precipitation. The increase in precipitation in the range of var. *villosa*, and to a large extend var. *sieberiana*, may be responsible for the future expansion of their suitable habitats. *Vachellia sieberiana* seeds have a thick coat that often requires mechanical damage and a comparatively higher amount of moisture for successful germination. Damage of the coat is mainly achieved by insect attack, especially the Bruchid beetles (*Bruchidius* spp.), and by digestion in the alimentary canal of herbivores (Mucunguzi & Oryem‐Origa, 1996; Mugunga & Sahinkuye, 2020; Sabiiti & Wein, 1987). The relatively large size of the *V. sieberiana* seeds means a smaller surface area is in contact with the soil for adequate water imbibition and, therefore, a higher moisture content is necessary for successful germination. It has been shown that germination percentage of *V. sieberiana* seeds increases with increasing moisture (Matekaire & Maroyi, 2007; Mugunga & Sahinkuye, 2020). Shoot biomass, height of seedlings, number of leaves and leaflets, and number of thorns were also shown to be positively correlated with watering intensity in *V. sieberiana* (Mugunga & Sahinkuye, 2020). The positive responses to watering intensity suggest that *V. sieberiana* is poorly adapted to water stress (Matekaire & Maroyi, 2007), and hence the contraction of the suitable habitat of var. *woodii* in a region predicted to receive less precipitation. Conservation and management practices of the three varieties should, therefore, be unique and considerate of the different responses to climate change.

## 5 CONCLUSIONS

Ensemble SDMs incorporating intraspecific information showed that the suitable habitat of *V. sieberiana* var. *sieberiana* predominantly occurs in the Sudanian and Zambezian phytochoria while that of var. *villosa* occurs between the African equatorial rain forest and the Sahel. It was also shown that the suitable habitat of var. *woodii* largely occurs in the Zambezian phytochorion. There is coexistence of var. *sieberiana* and var. *villosa* in the Sudanian phytochorion, and of var. *sieberiana* and var. *woodii* in the Zambezian phytochorion. Due to the coexistence, it is vital and necessary in studies of *V. sieberiana* to identify the species to variety level. Ensemble SDMs also revealed different responses of the three varieties of *V. sieberiana* to climate change. The suitable habitat of var. *woodii* was predicted to contract in a region likely to receive low precipitation. On the other hand, the suitable habitats of var. *villosa* and var. *sieberiana* were predicted to expand in regions expected to receive high precipitation in the near and long term. The contraction under water stress and expansion under high moisture content suggest the varieties are poorly adapted to withstand water stress. Incorporating intraspecific information in SDMs has revealed cryptic responses of intraspecific taxa to climate change that would otherwise have not been detected by a species‐level SDM.

## AUTHOR CONTRIBUTIONS

**Percy Jinga:** Conceptualization (lead); data curation (lead); formal analysis (lead); software (lead); visualization (supporting); writing – original draft (lead); writing – review and editing (equal). **Tawanda Manyangadze:** Conceptualization (supporting); data curation (supporting); formal analysis (supporting); visualization (lead); writing – review and editing (equal).

## CONFLICT OF INTEREST STATEMENT

The authors have no relevant financial or non‐financial interests to disclose.

## Supporting information


Appendix S1

Appendix S2

Appendix S3

Appendix S4

## Data Availability

Occurrence records of the three varieties of *V. sieberiana* from GBIF are publicly available at https://doi.org/10.15468/dl.sn42cb (var. *woodii*), https://doi.org/10.15468/dl.fwmmv4 (var. *villosa*), and https://doi.org/10.15468/dl.8fxhvq (var. *sieberiana*), while those from the RAINBIO database are publicly available at https://gdauby.github.io/rainbio/index.html. Environmental variables are available at https://worldclim.org.
