# Supplementary material for: Variable intraspecific response to climate change in a medicinally important African tree species, Vachellia sieberiana (DC.) (paperbark thorn)
Source: Ecol Evol. 2024 Apr 29;14(5):e11314. doi: 10.1002/ece3.11314 (PMC11056962; doi:10.1002/ece3.11314)

**Variable intraspecific response to climate change in a medicinally important Africa tree species, *Vachellia sieberiana* (DC.) (Paperbark thorn)**

**Appendix S1**

Location of occurrence records of *Vachellia sieberiana* used in ensemble species distribution models

(a) = *V. sieberiana* var. *sieberiana*

(b) = *V. sieberiana* var. *villosa*

(c) = *V. sieberiana* var. *woodii*

(c)

(b)

(a)


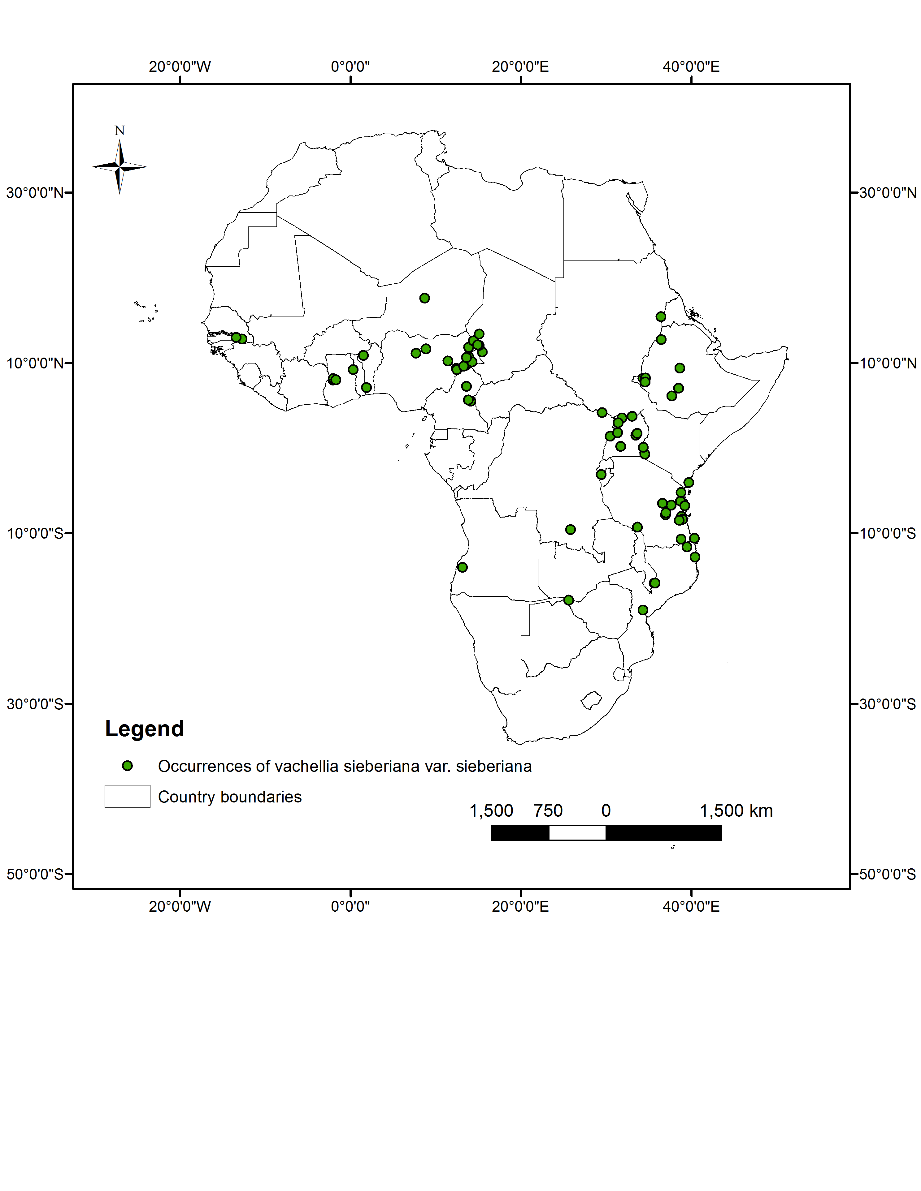

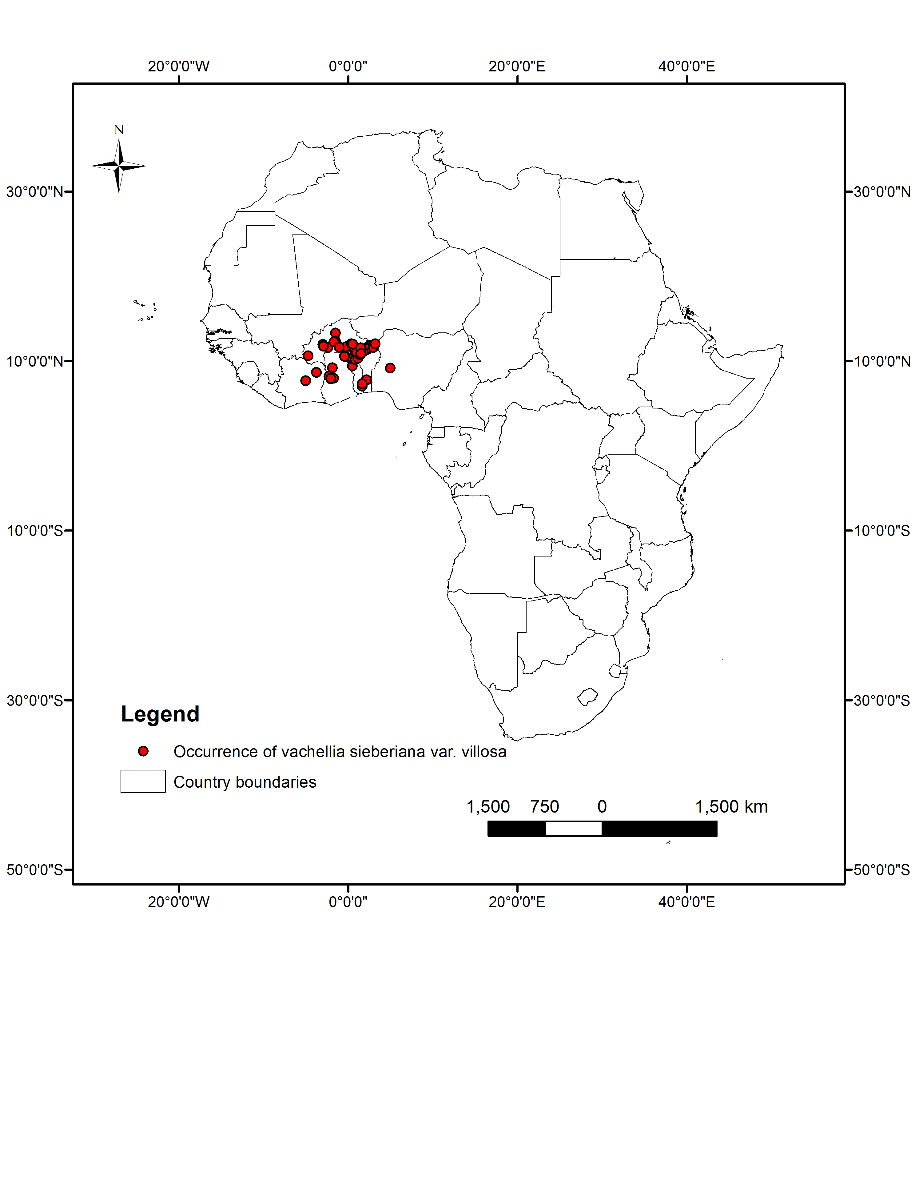

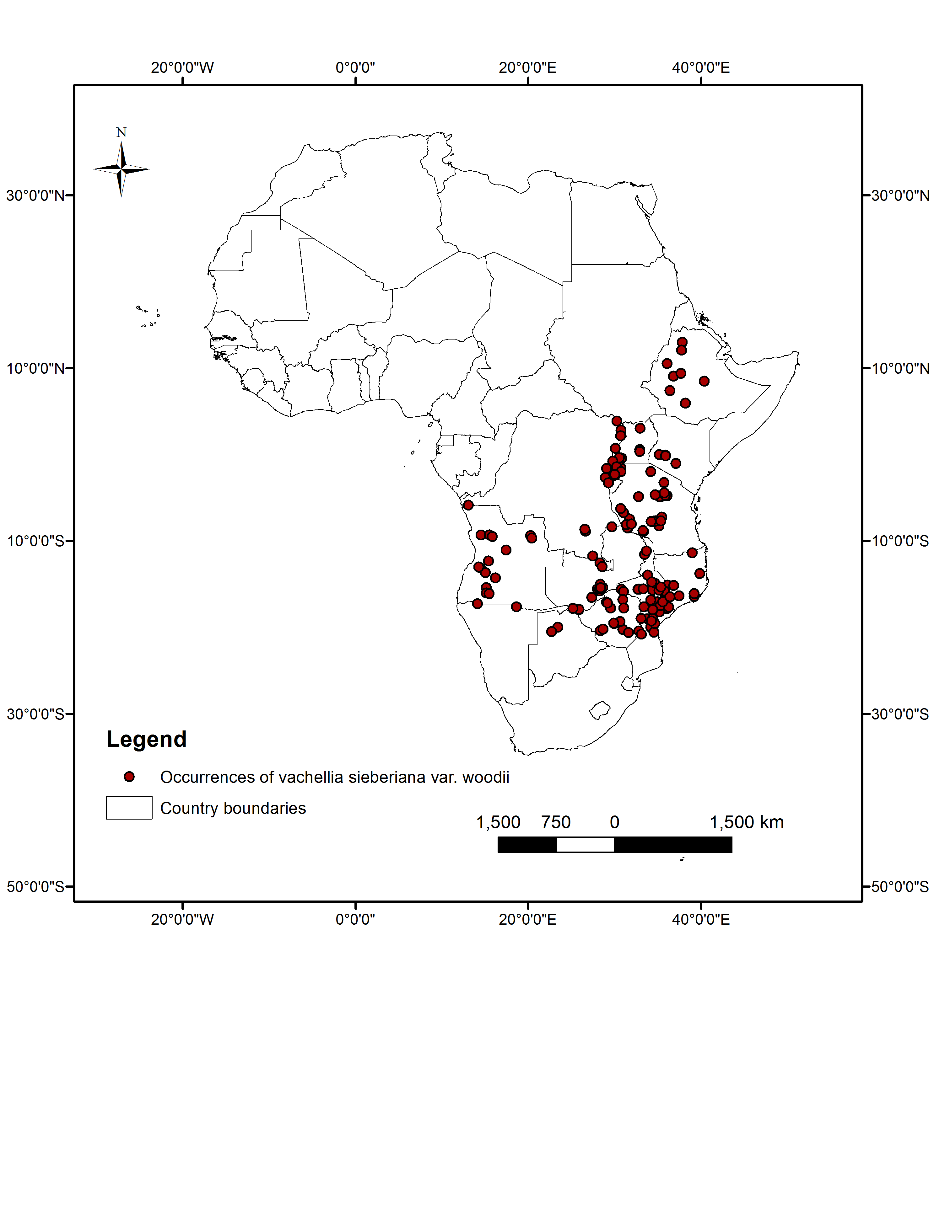

Supplement: Supplementary file 1 — Appendix S1 [file ECE3-14-e11314-s001.docx]
