# Supplementary material for: Variable intraspecific response to climate change in a medicinally important African tree species, Vachellia sieberiana (DC.) (paperbark thorn)
Source: Ecol Evol. 2024 Apr 29;14(5):e11314. doi: 10.1002/ece3.11314 (PMC11056962; doi:10.1002/ece3.11314)
Supplement: Supplementary file 2 — Appendix S2 [file ECE3-14-e11314-s004.docx]

**Variable intraspecific response to climate change in a medicinally important African tree species, *Vachellia sieberiana* (DC.) (Paperbark thorn)**

**Appendix S2**

1. Response curves of variables used in an ensemble species distribution model to estimate the current suitable habitat of *Vachellia sieberiana* var. *sieberiana*


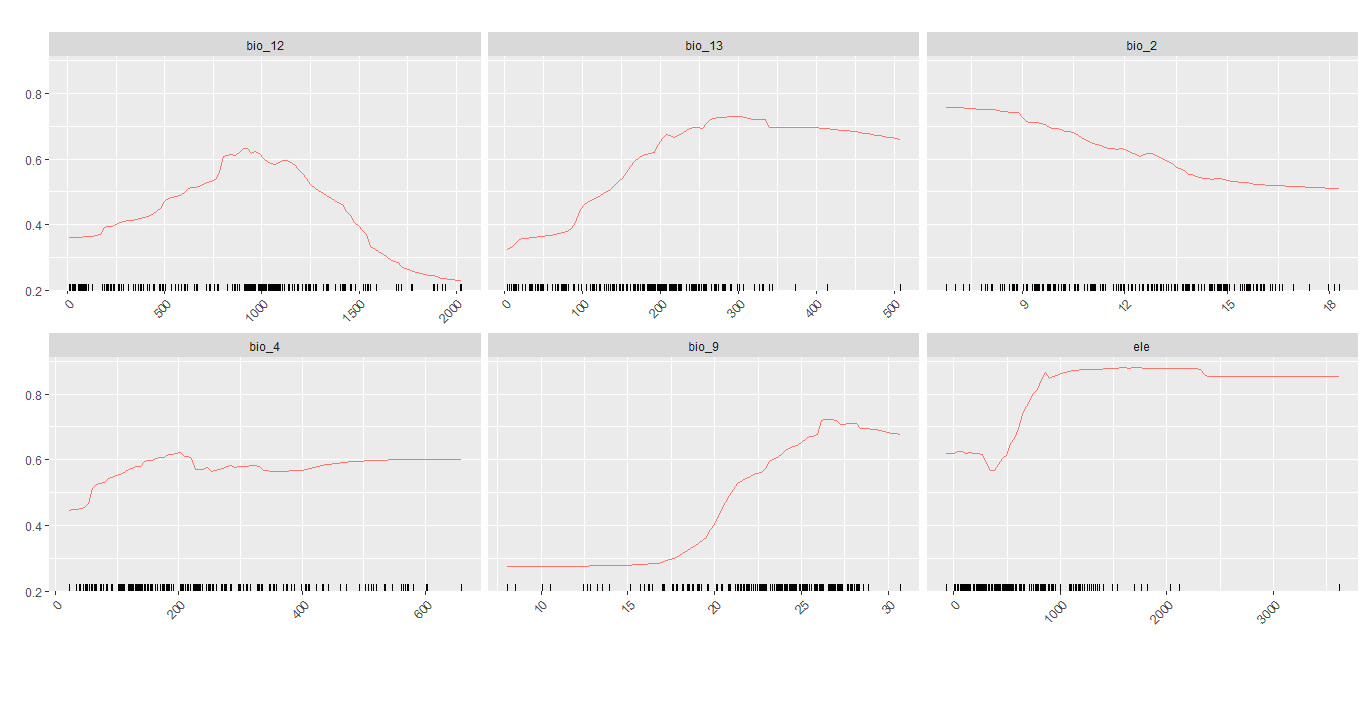


bio_2 = annual mean temperature

bio_4 = temperature seasonality

bio_9 = mean temperature of driest quarter

bio_12 = annual precipitation

bio_13 = precipitation of wettest quarter

ele = elevation

2. Response curves of variables used in an ensemble species distribution model to estimate the current suitable habitat of *Vachellia sieberiana* var. *villosa*


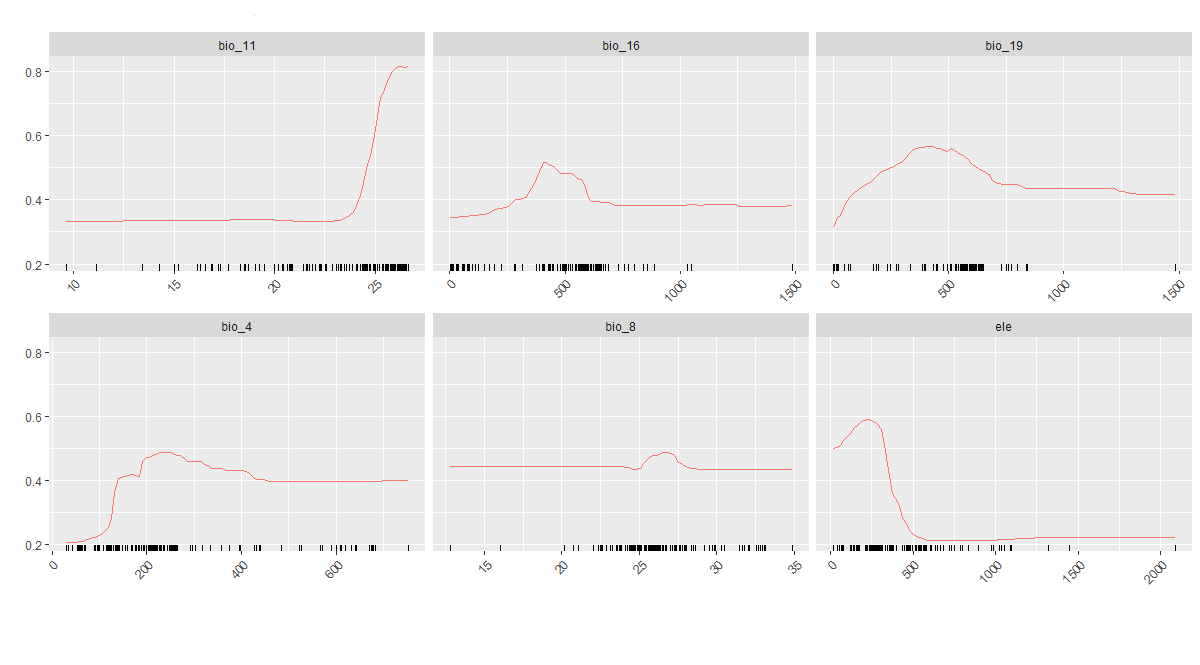


bio_4 = temperature seasonality

bio_8 = mean temperature of wettest quarter

bio_11 = mean temperature of coldest quarter

bio_16 = precipitation of wettest quarter

bio_19 = precipitation of coldest quarter

ele = elevation

3. Response curves of variables used in an ensemble species distribution model to estimate the current suitable habitat of *Vachellia sieberiana* var. *woodii*


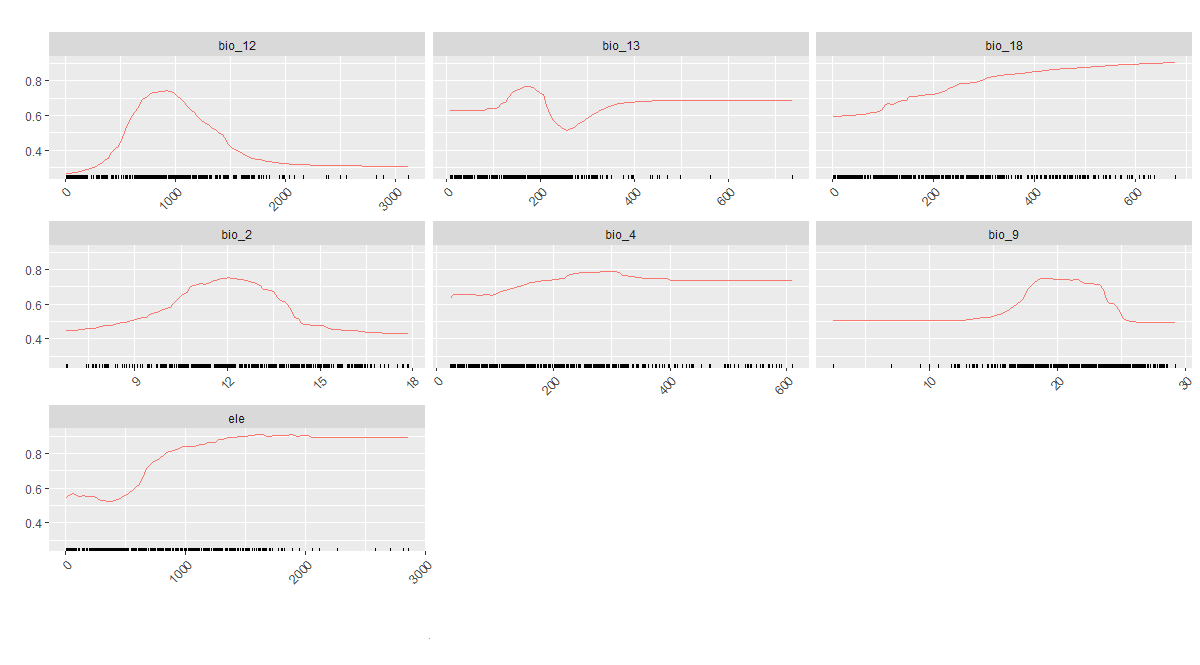


bio_2 = annual mean temperature

bio_4 = temperature seasonality

bio_9 = mean temperature of driest quarter

bio_12 = annual precipitation

bio_13 = precipitation of wettest quarter

bio_18 = precipitation of warmest quarter

ele = elevation
