# Supplementary material for: Variable intraspecific response to climate change in a medicinally important African tree species, Vachellia sieberiana (DC.) (paperbark thorn)
Source: Ecol Evol. 2024 Apr 29;14(5):e11314. doi: 10.1002/ece3.11314 (PMC11056962; doi:10.1002/ece3.11314)
Supplement: Supplementary file 3 — Appendix S3 [file ECE3-14-e11314-s003.docx]

**Variable intraspecific response to climate change in a medicinally important African tree species, *Vachellia sieberiana* (DC.) (Paperbark thorn)**

**Appendix S3**

Percentage changes in the size of suitable habitat of three varieties of *Vachellia sieberiana* under three general circulation models (GCMs), two shared socio-economic pathways (SSPs) and two time periods

|  |  |  | **CanESM5** | **IPSL-CM6A-LR** | **MIROC6** | **Mean** |
| --- | --- | --- | --- | --- | --- | --- |
| **SSP2-4.5** | **2041-2060** | Var. *sieberiana* | 36.992 | 33.533 | 30.95 | 33.825 |
|  |  | Var. *villosa* | 159.736 | 116.887 | 82.331 | 119.6513 |
|  |  | Var. *woodii* | -14.832 | -2.62 | -7.621 | -8.35767 |
|  | **2081-2100** | Var. *sieberiana* | 39.065 | 43.291 | 39.502 | 40.61933 |
|  |  | Var. *villosa* | 187.366 | 149.986 | 122.761 | 153.371 |
|  |  | Var. *woodii* | -20.349 | -10.444 | -12.466 | -14.4197 |
|  |  |  |  |  |  |  |
| **SSP5-8.5** | **2041-2060** | Var. *sieberiana* | 33.098 | 40.228 | 42.474 | 38.6 |
|  |  | Var. *villosa* | 174.764 | 132.581 | 109.605 | 138.9833 |
|  |  | Var. *woodii* | -18.457 | -4.796 | -8.073 | -10.442 |
|  | **2081-2100** | Var. *sieberiana* | 39.251 | 56.521 | 52.13 | 49.30067 |
|  |  | Var. *villosa* | 281.172 | 222.976 | 196.122 | 233.4233 |
|  |  | Var. *woodii* | -42.074 | -25.01 | -25.099 | -30.7277 |
