# Supplementary material for: Variable intraspecific response to climate change in a medicinally important African tree species, Vachellia sieberiana (DC.) (paperbark thorn)
Source: Ecol Evol. 2024 Apr 29;14(5):e11314. doi: 10.1002/ece3.11314 (PMC11056962; doi:10.1002/ece3.11314)
Supplement: Supplementary file 4 — Appendix S4 [file ECE3-14-e11314-s002.docx]

**Variable intraspecific response to climate change in a medicinally important African tree species, *Vachellia sieberiana* (DC.) (Paperbark thorn)**

**Appendix S4**

Predicted distributions of three varieties of *Vachellia sieberiana* under different climate change scenarios

**1. SSP2-4.5**

(a) 2041-2060


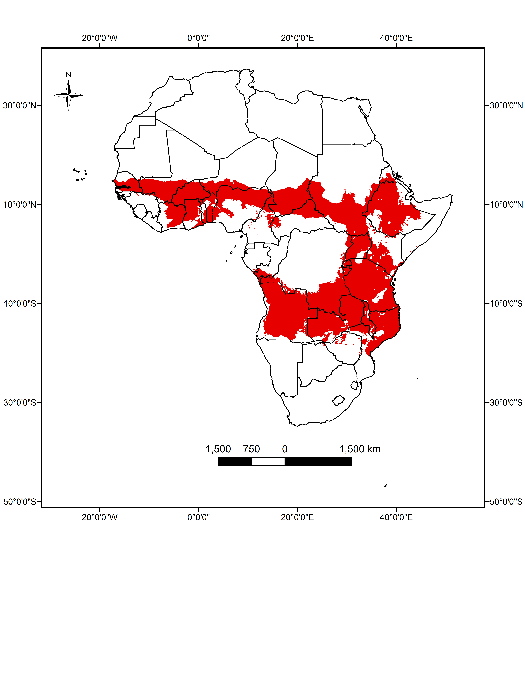

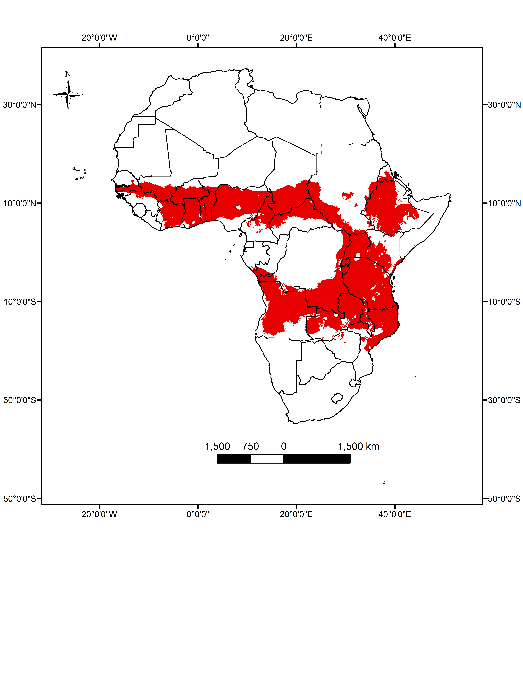

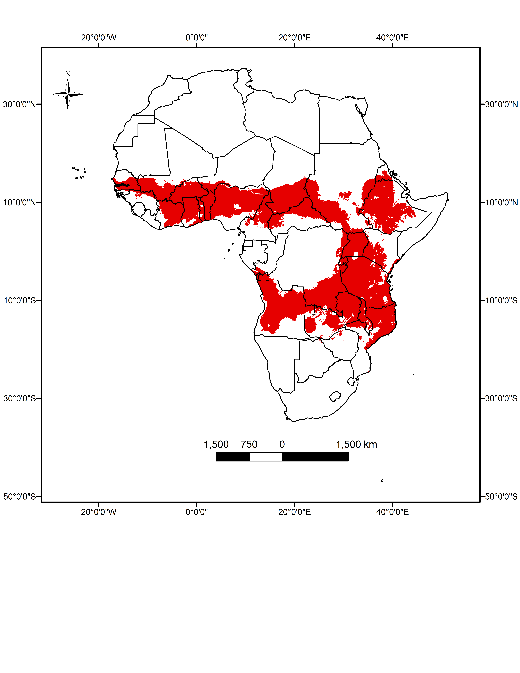

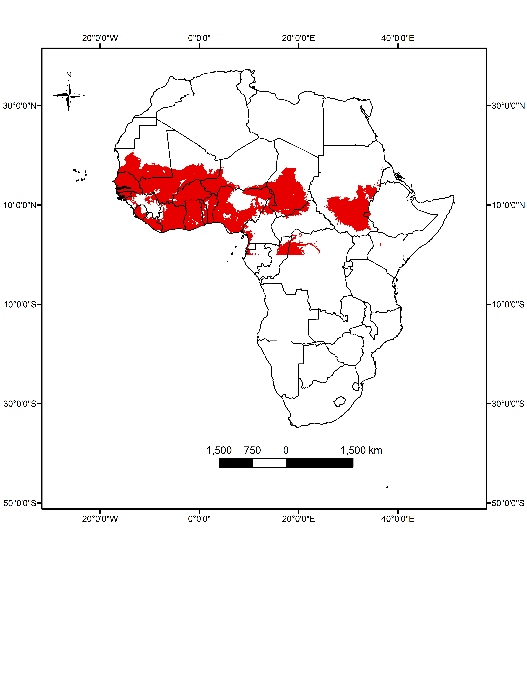

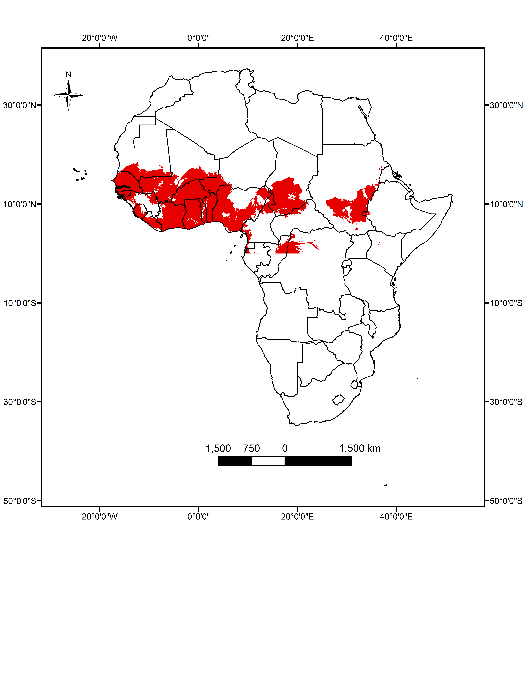

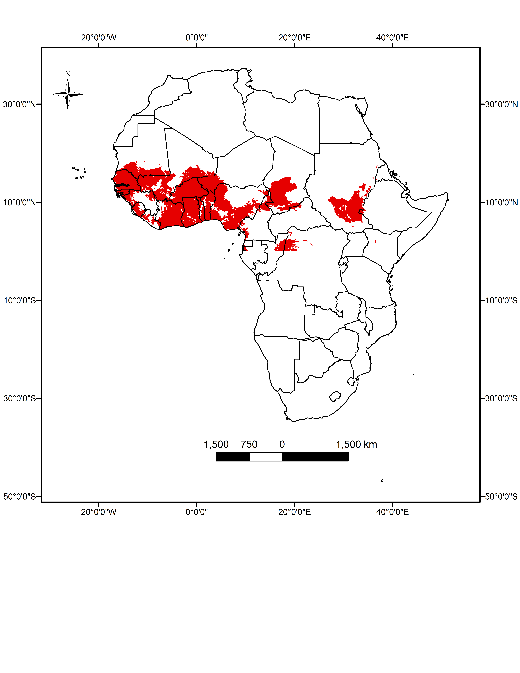

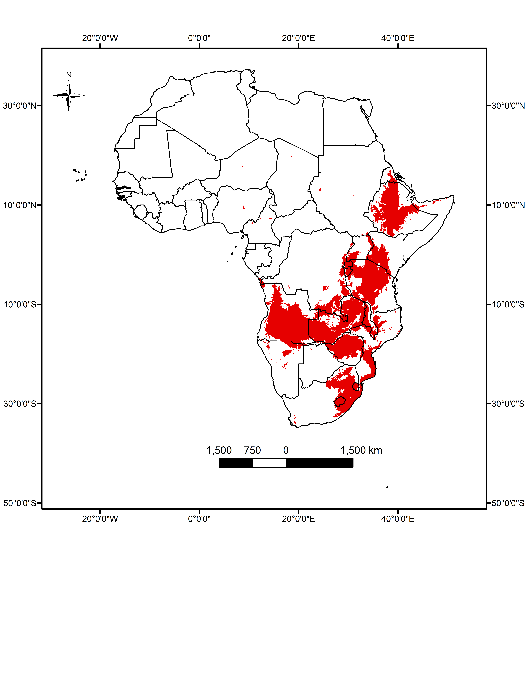

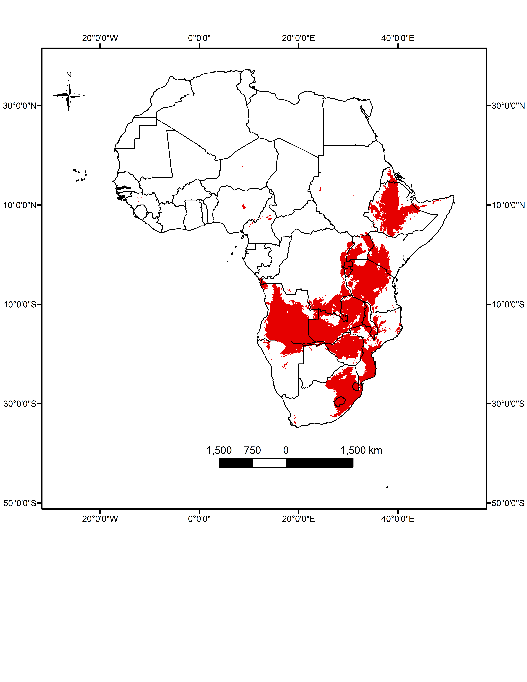

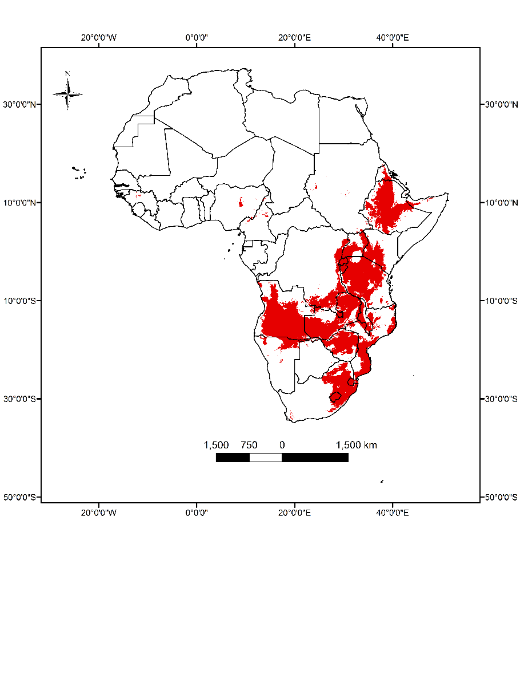


Column 1 = CanESM5, Column 2 = IPSL-CM6A-LR, Column 3 = MIROC6

Row 1 = var. *sieberiana*, Row 2 = var. *villosa*, Row 3 = var. *woodii*

(b) 2081-2100


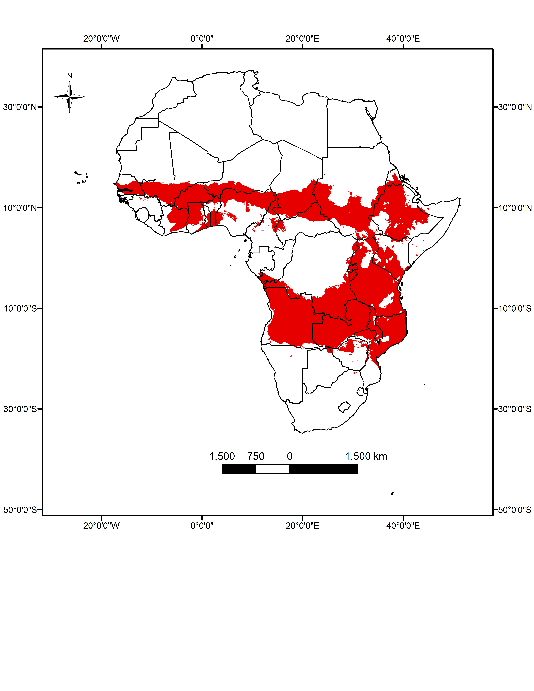

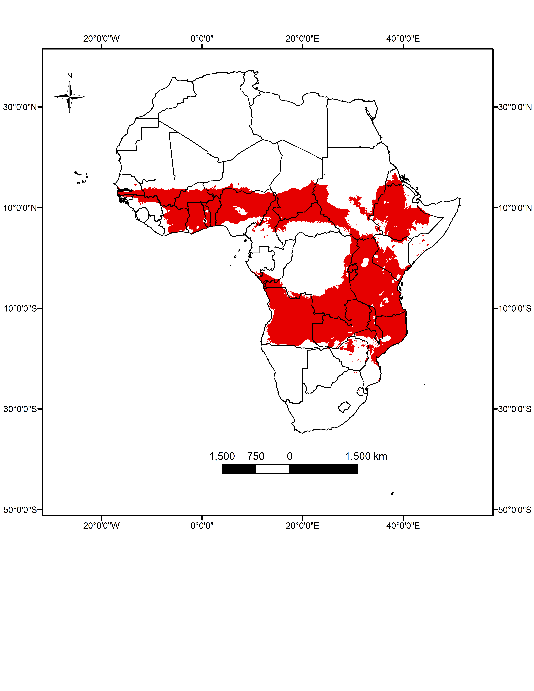

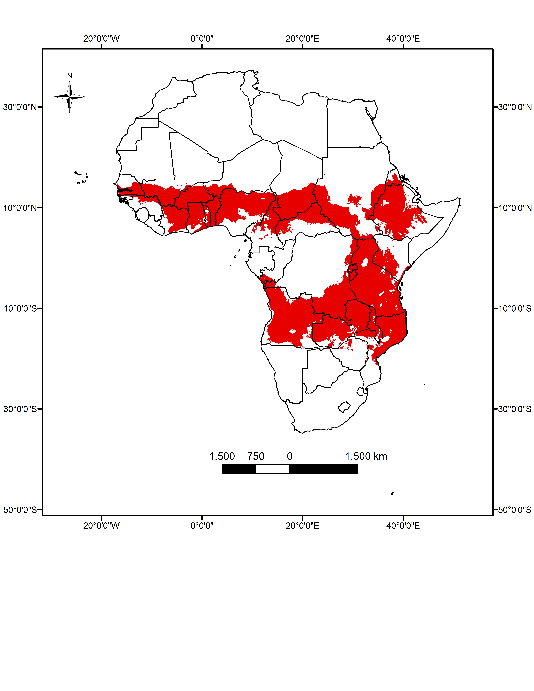

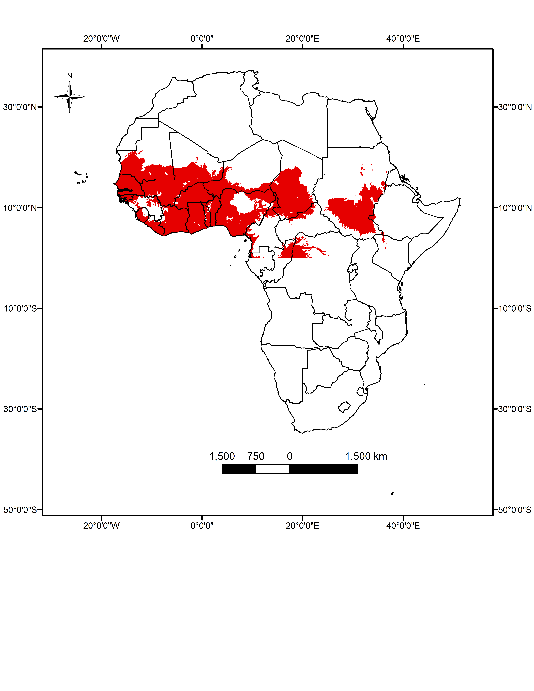

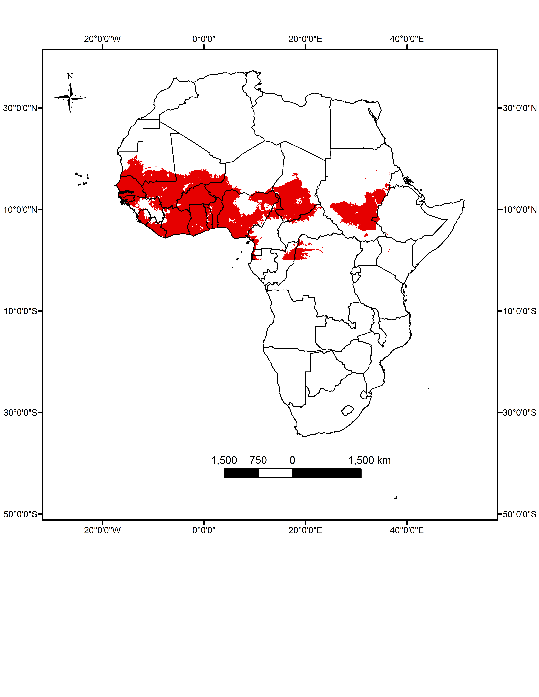

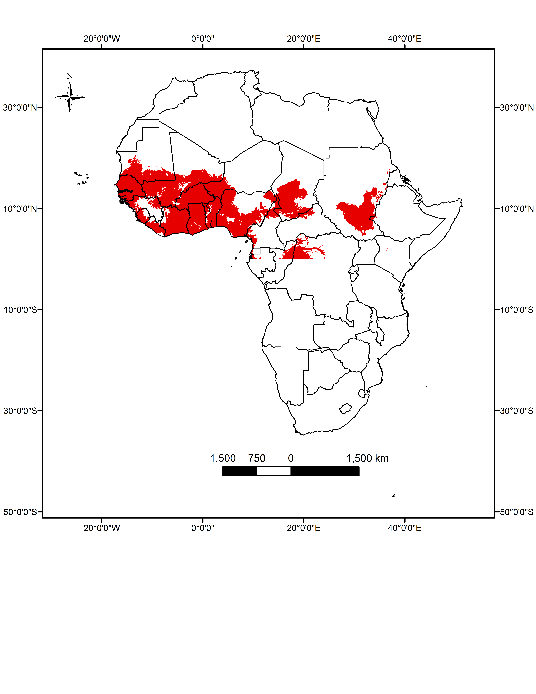

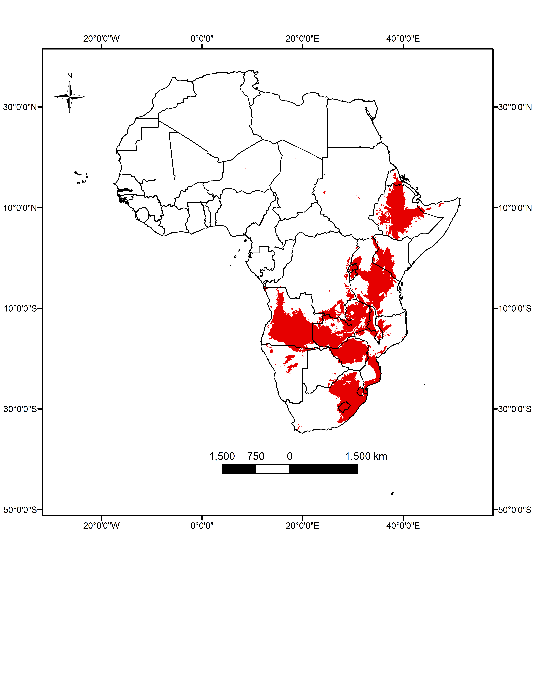

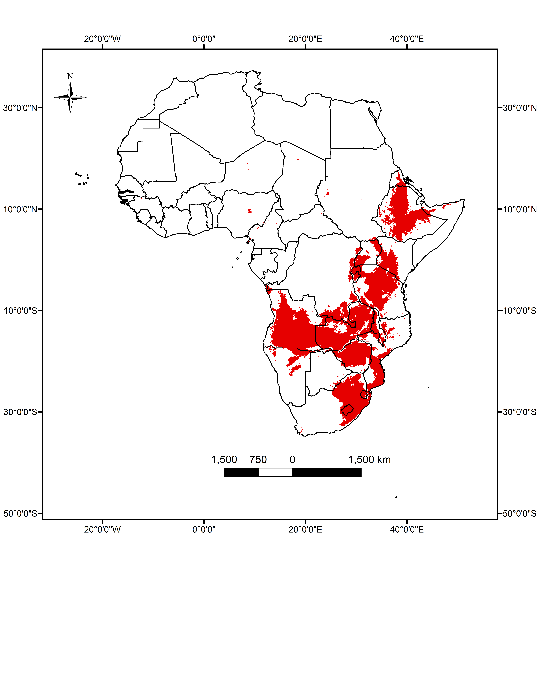

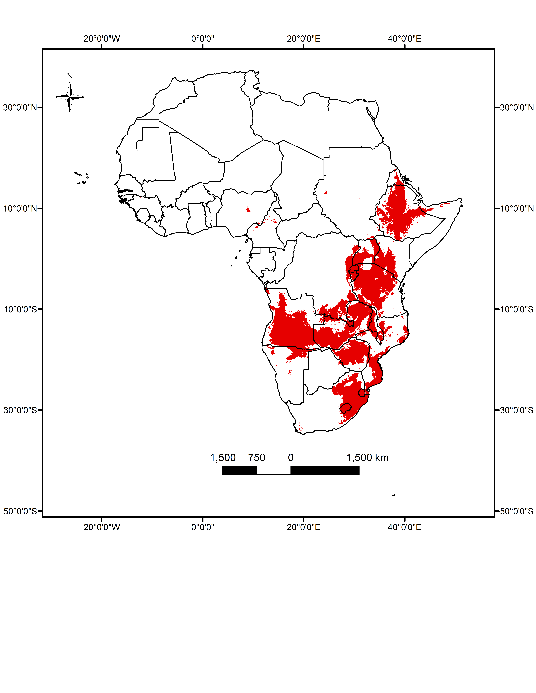


Column 1 = CanESM5, Column 2 = IPSL-CM6A-LR, Column 3 = MIROC6

Row 1 = var. *sieberiana*, Row 2 = var. *villosa*, Row 3 = var. *woodii*

**2. SSP5-8.5**

(a) 2041-2060


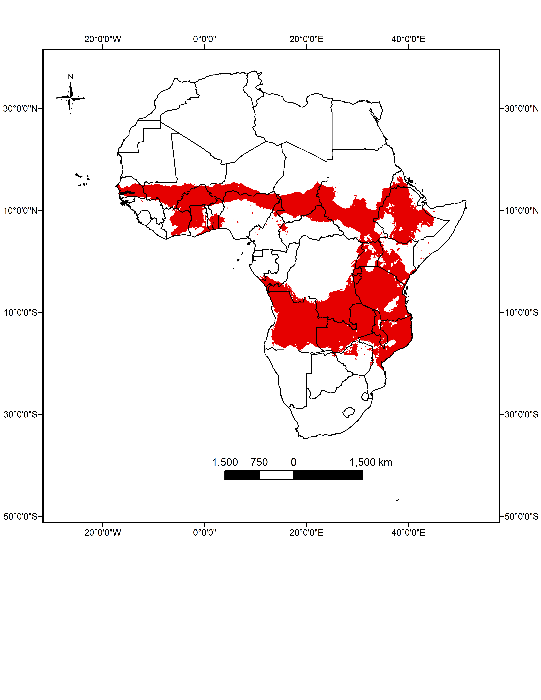

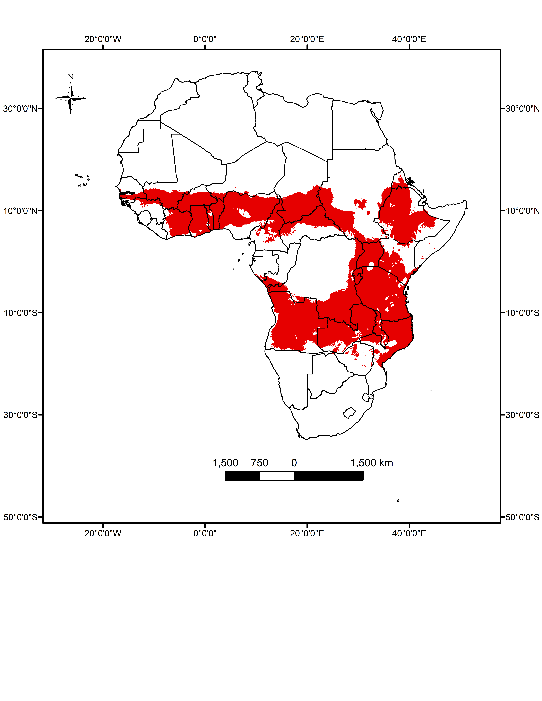

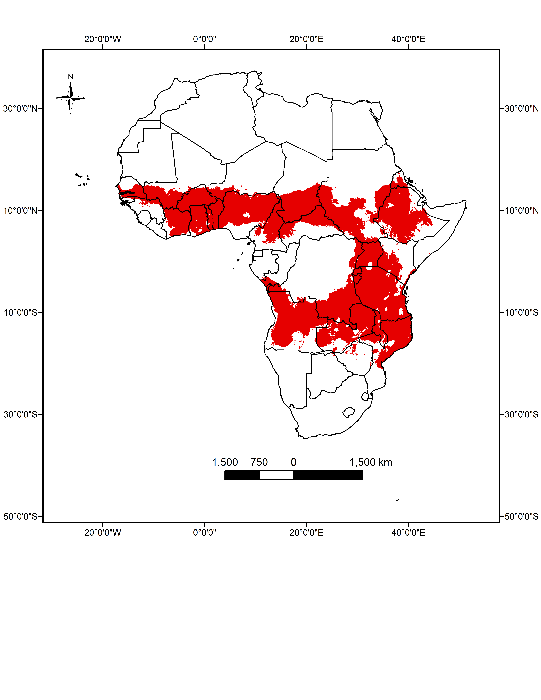

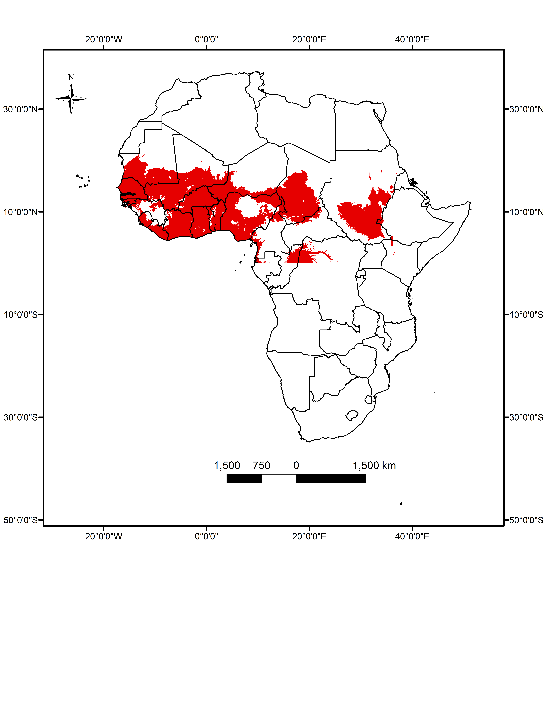

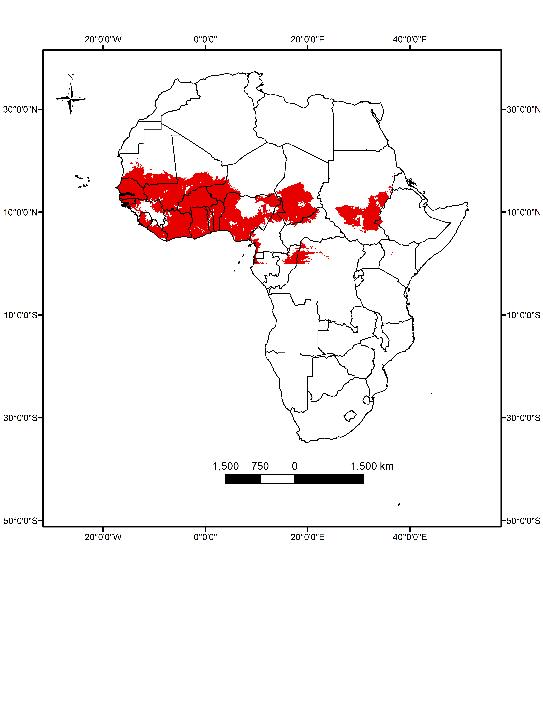

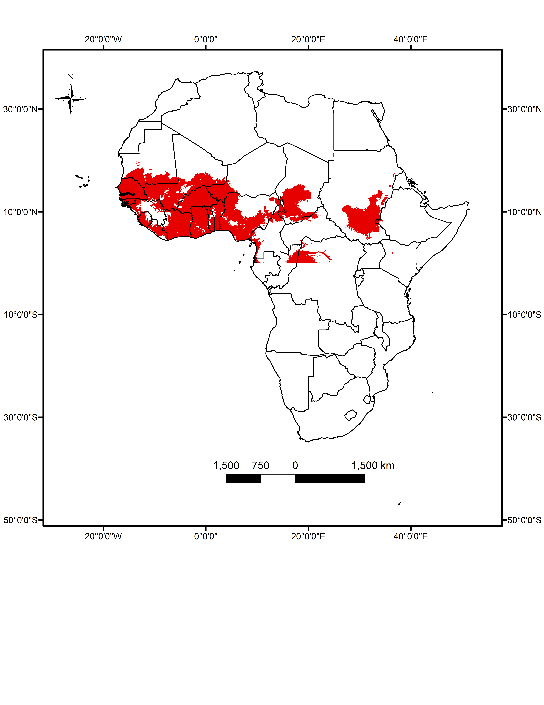

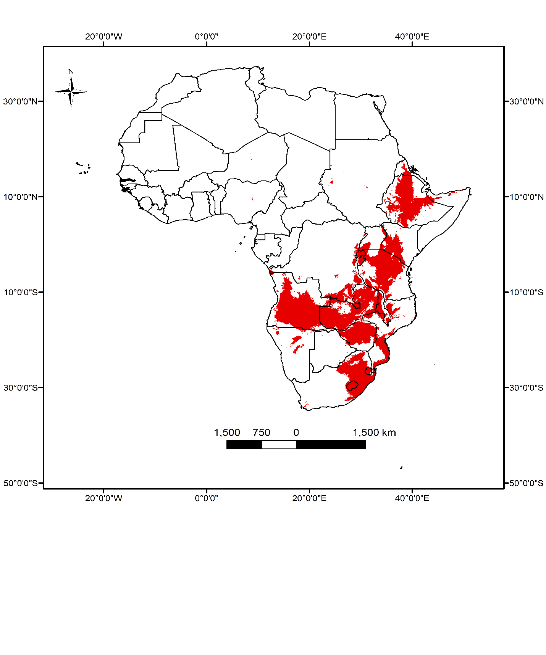

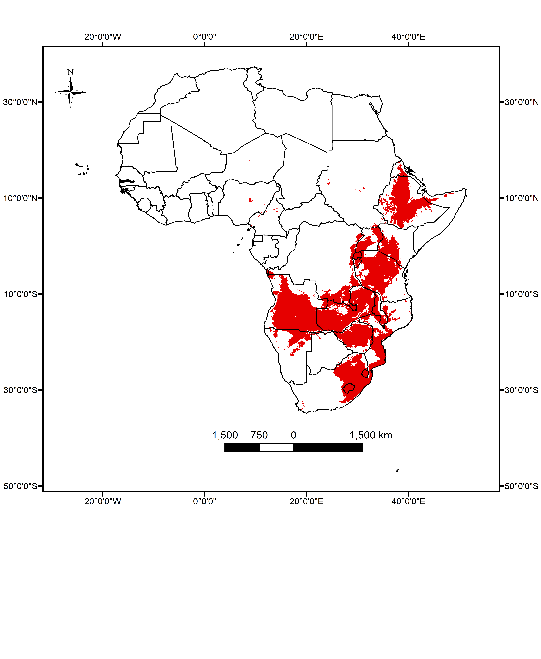

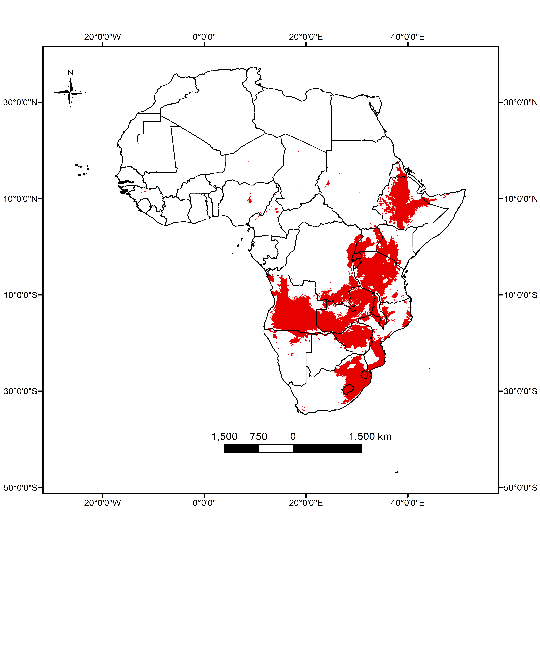


Column 1 = CanESM5, Column 2 = IPSL-CM6A-LR, Column 3 = MIROC6

Row 1 = var. *sieberiana*, Row 2 = var. *villosa*, Row 3 = var. *woodii*

(b) 2081-2100


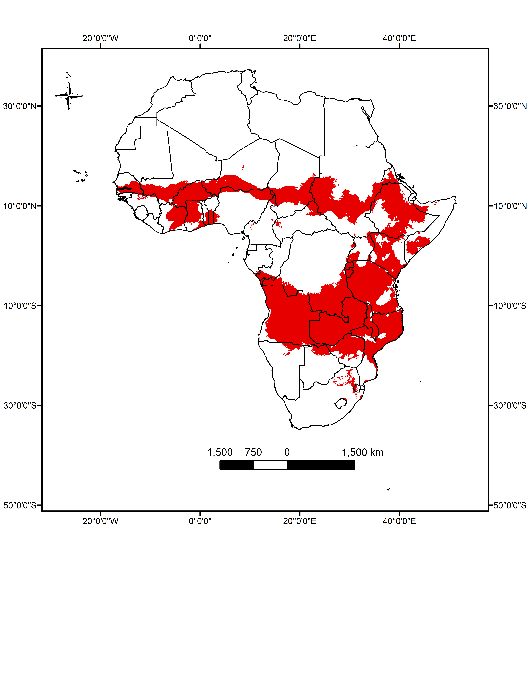

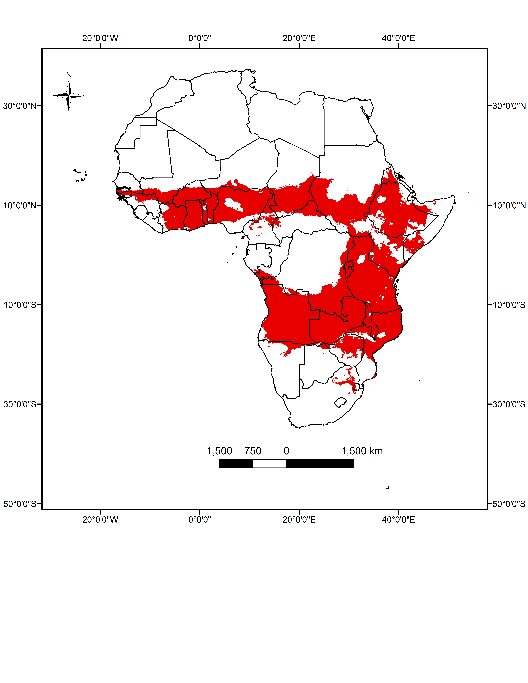

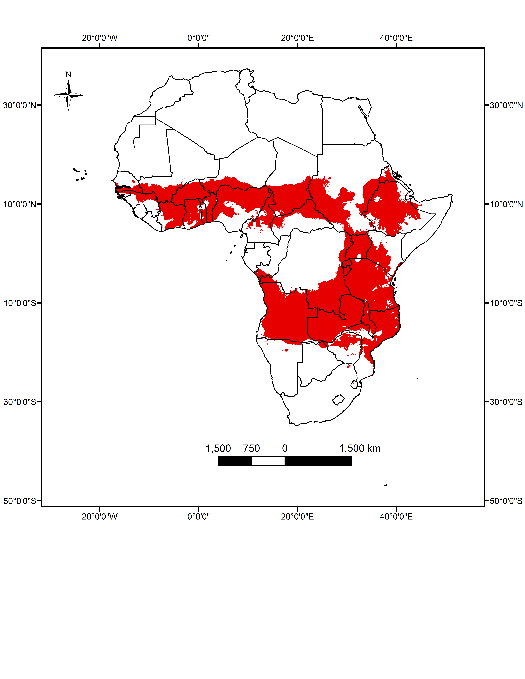

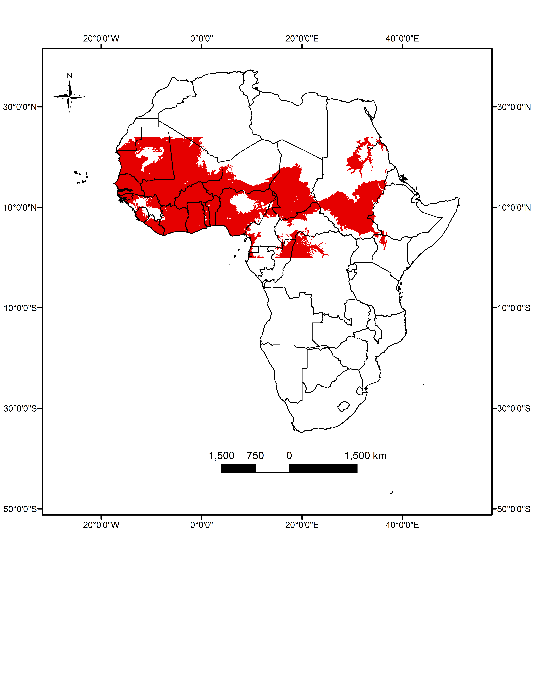

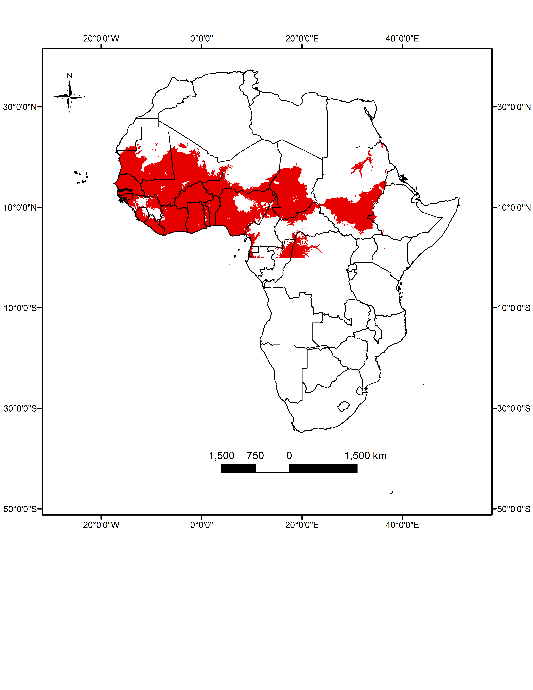

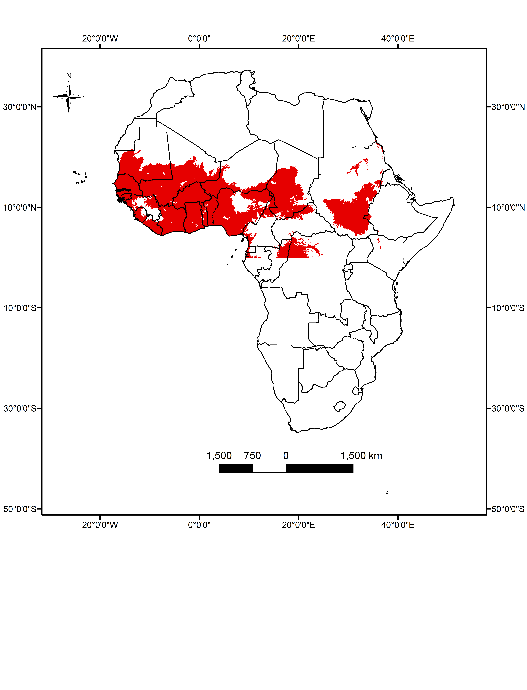

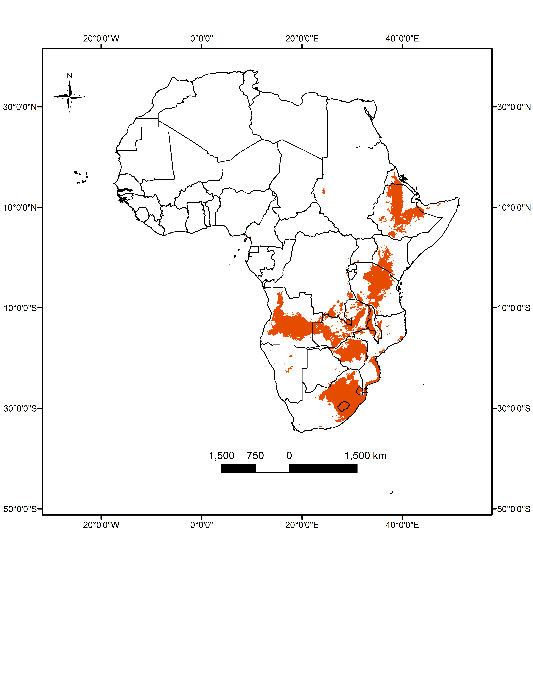

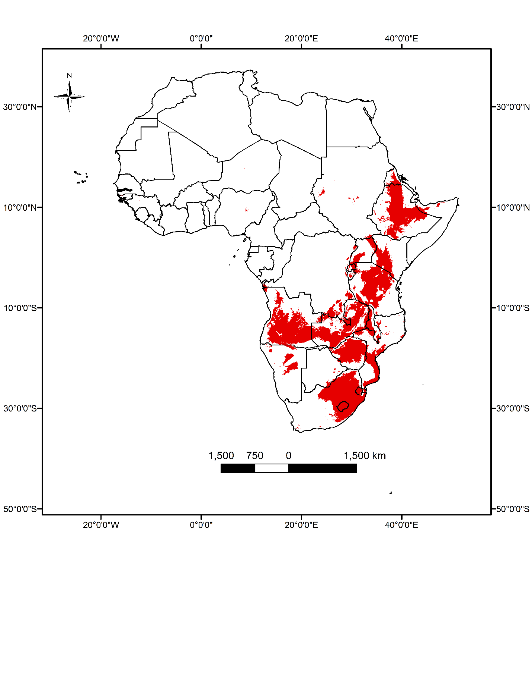

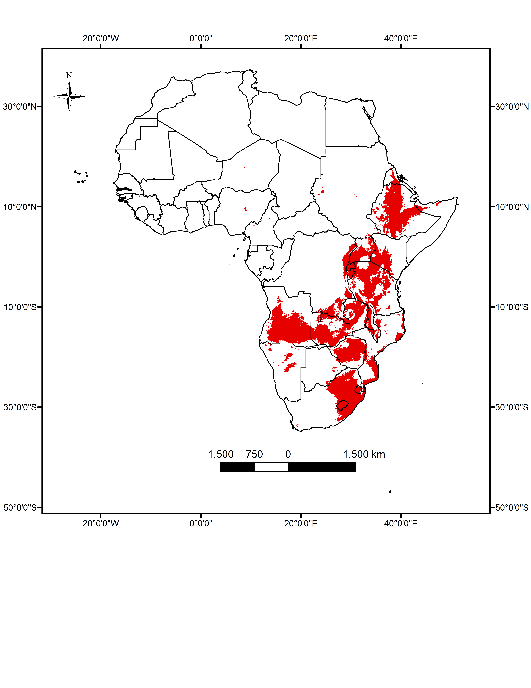


Column 1 = CanESM5, Column 2 = IPSL-CM6A-LR, Column 3 = MIROC6

Row 1 = var. *sieberiana*, Row 2 = var. *villosa*, Row 3 = var. *woodii*
